# Supplementary figures and images for: A back-door insight into the modulation of Src kinase activity by the polyamine spermidine
Source: eLife. 2023 Jun 30;12:e85872. doi: 10.7554/eLife.85872 (PMC10328509; doi:10.7554/eLife.85872)

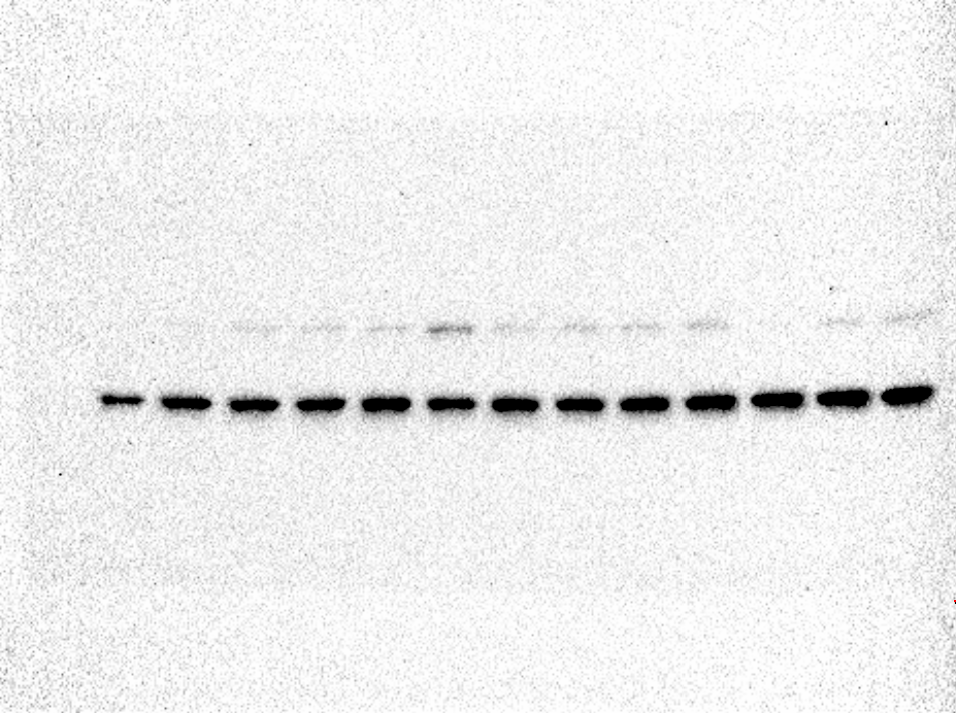

Supplement: Figure 1—source data 1. — Figure with the uncropped blots with relevant bands clearly labeled are provided. [file elife-85872-fig1-data1.zip › Figure 1-source data 1/a-Actin.tif]

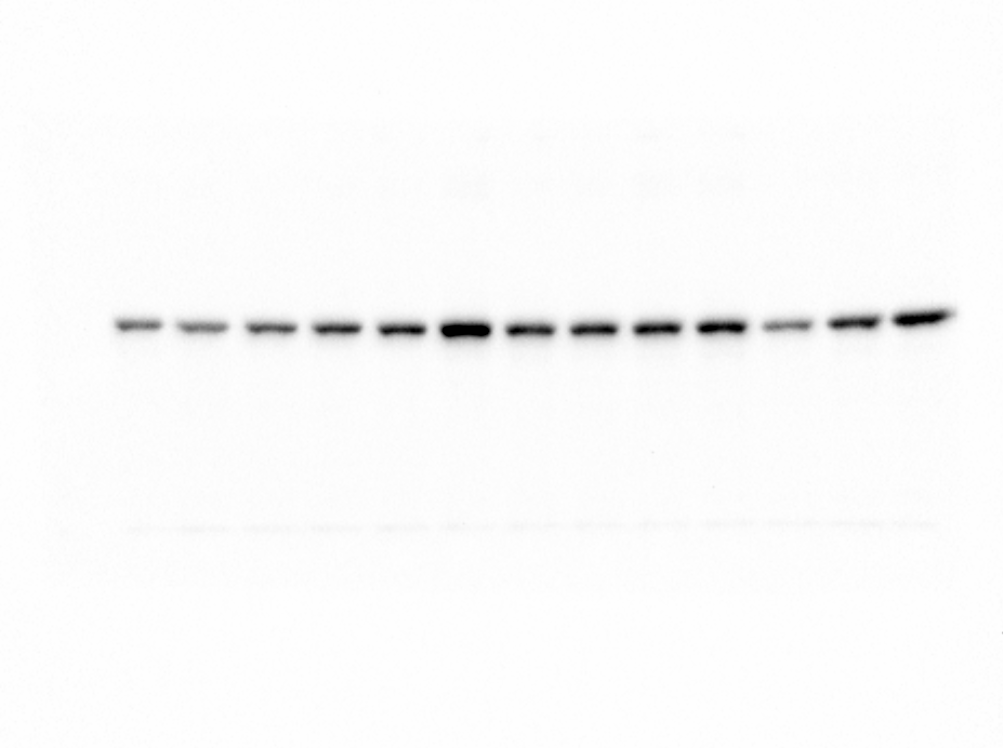

Supplement: Figure 1—source data 1. — Figure with the uncropped blots with relevant bands clearly labeled are provided. [file elife-85872-fig1-data1.zip › Figure 1-source data 1/a-pSRC.tif]

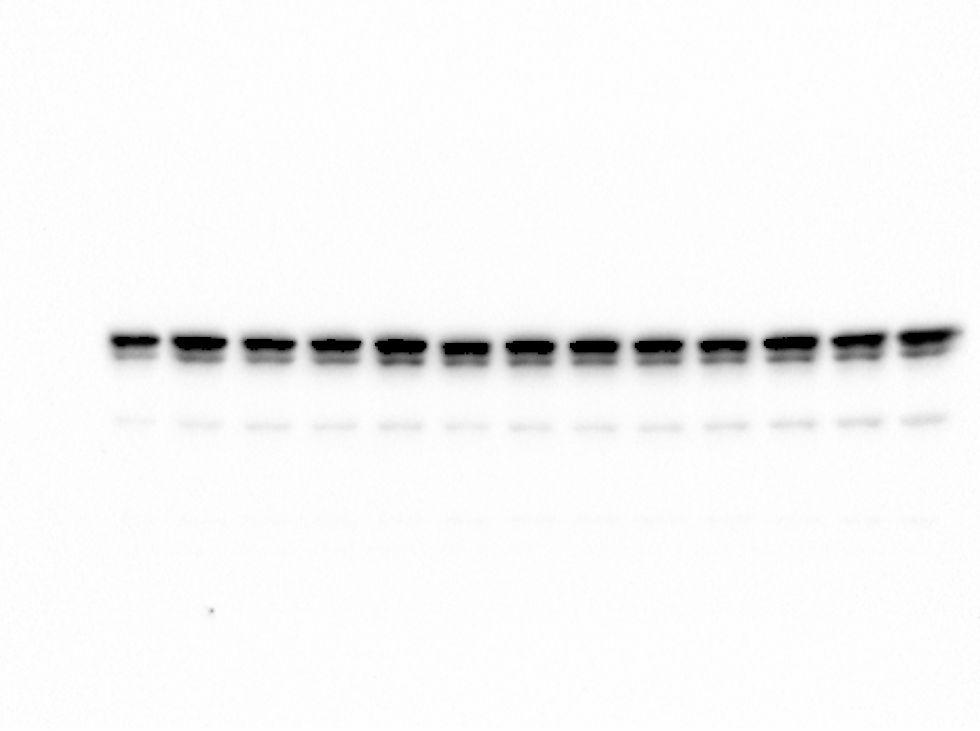

Supplement: Figure 1—source data 1. — Figure with the uncropped blots with relevant bands clearly labeled are provided. [file elife-85872-fig1-data1.zip › Figure 1-source data 1/a-Src.tif]

pSrc

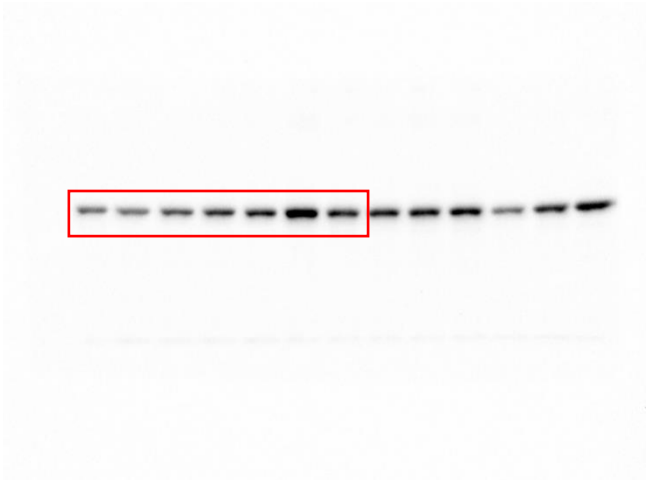

Src

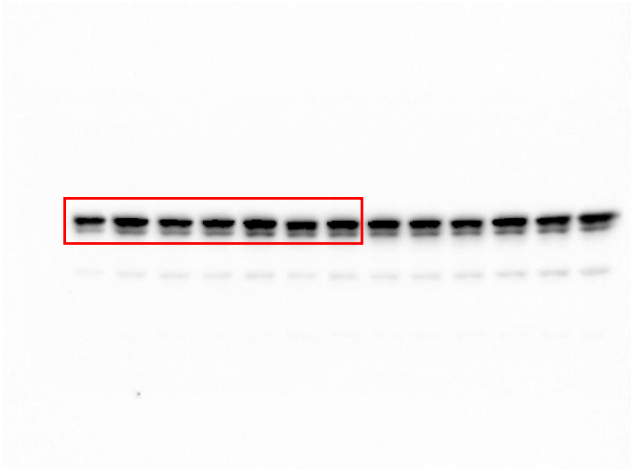

Actin

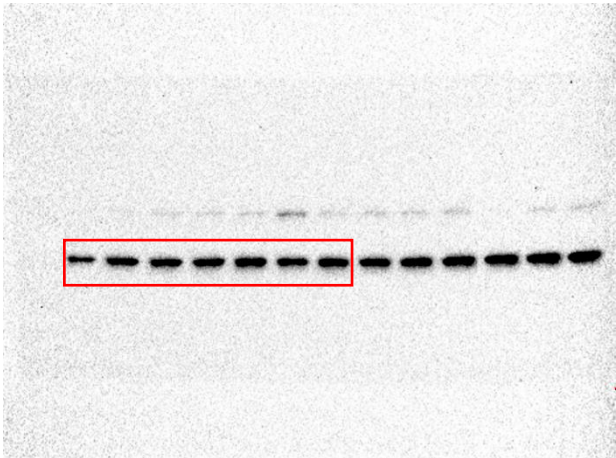

Supplement: Figure 1—source data 1. — Figure with the uncropped blots with relevant bands clearly labeled are provided. [file elife-85872-fig1-data1.zip › Figure 1-source data 1/Rossini et al. Figure 1B_uncropped.pdf]

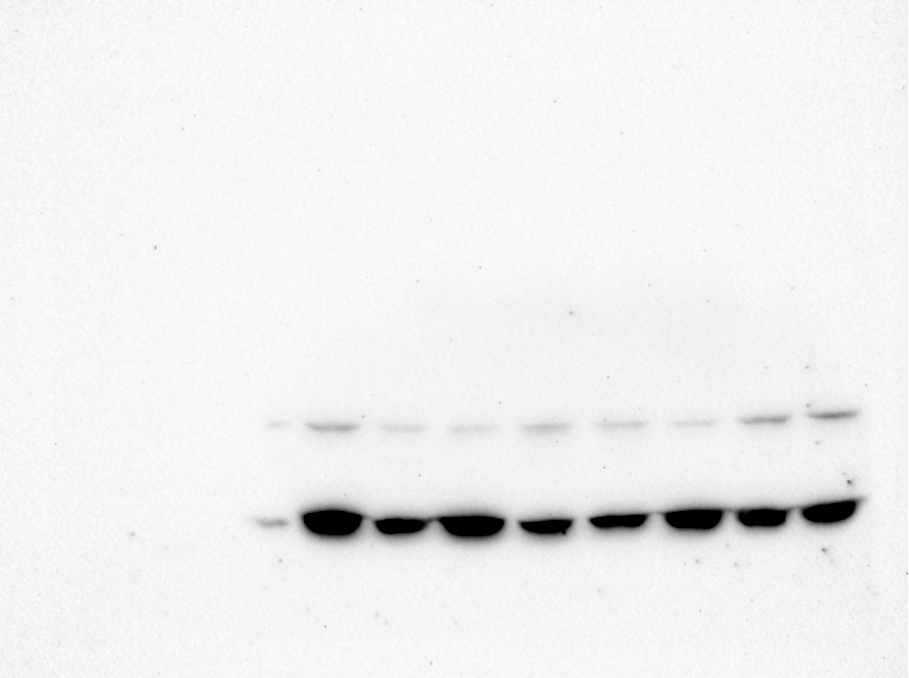

Supplement: Figure 1—source data 2. — Figure with the uncropped blots with relevant bands clearly labeled are provided. [file elife-85872-fig1-data2.zip › Figure 1-source data 2/a-actin.tif]

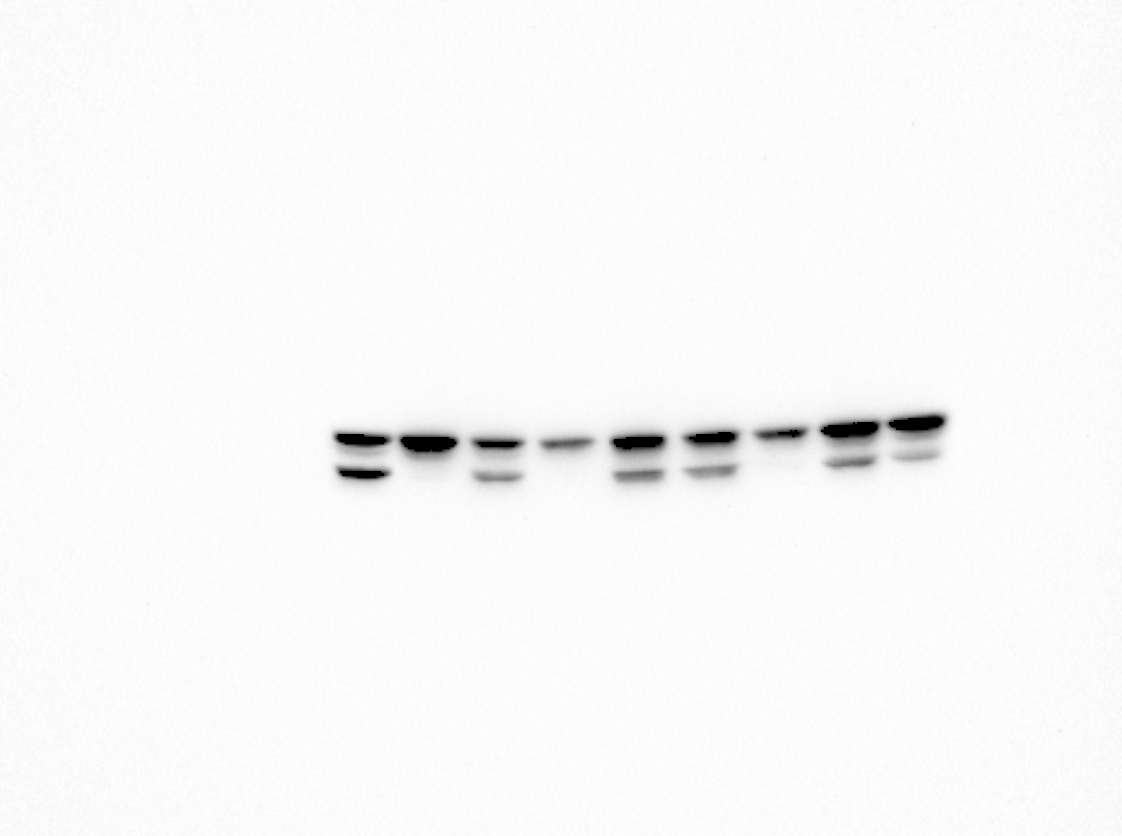

Supplement: Figure 1—source data 2. — Figure with the uncropped blots with relevant bands clearly labeled are provided. [file elife-85872-fig1-data2.zip › Figure 1-source data 2/a-pSRC.tif]

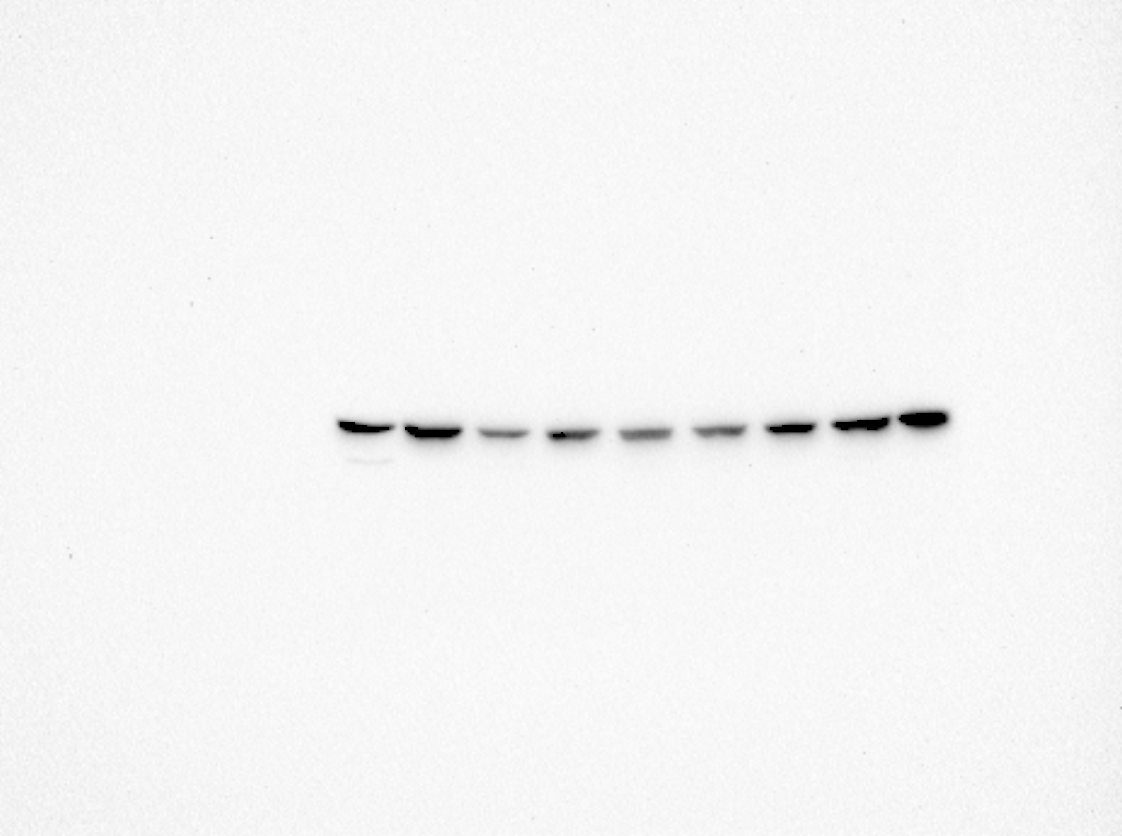

Supplement: Figure 1—source data 2. — Figure with the uncropped blots with relevant bands clearly labeled are provided. [file elife-85872-fig1-data2.zip › Figure 1-source data 2/a-SRC.tif]

Rossini et al. Raw Figure 1D (revised)

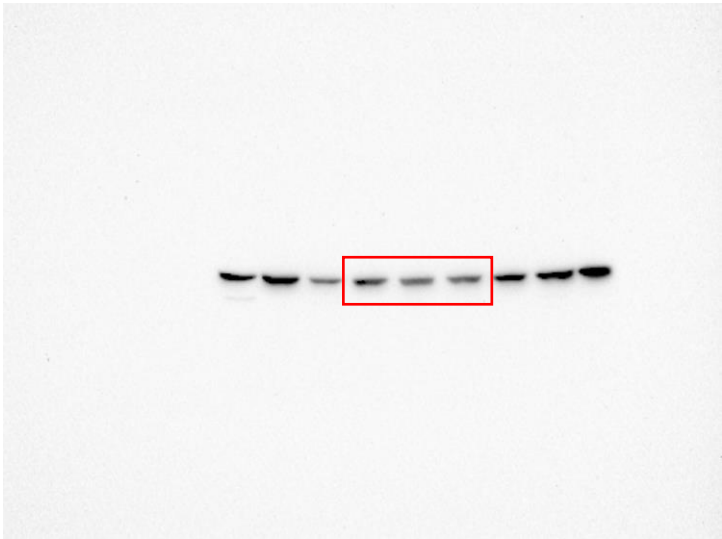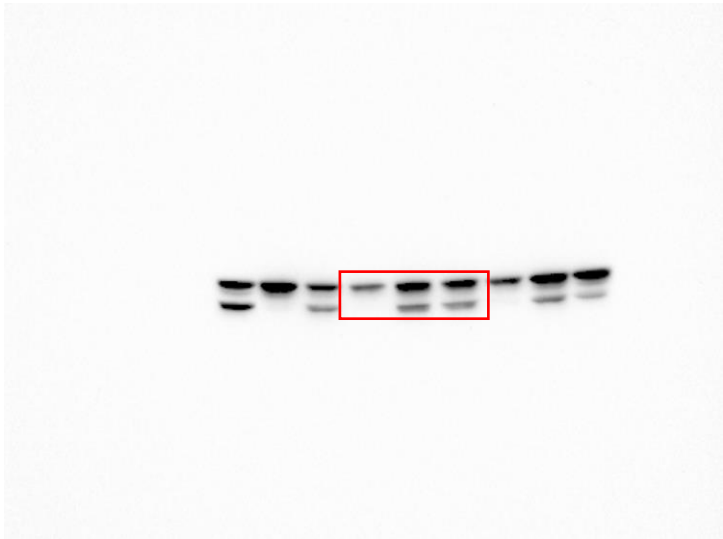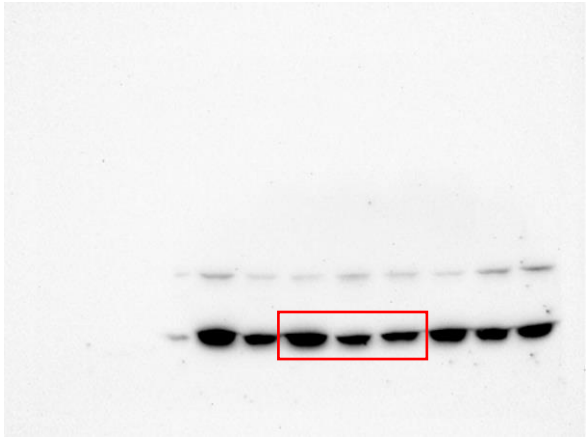

Supplement: Figure 1—source data 2. — Figure with the uncropped blots with relevant bands clearly labeled are provided. [file elife-85872-fig1-data2.zip › Figure 1-source data 2/Rossini et al. Figure 1D-uncropped.pdf]

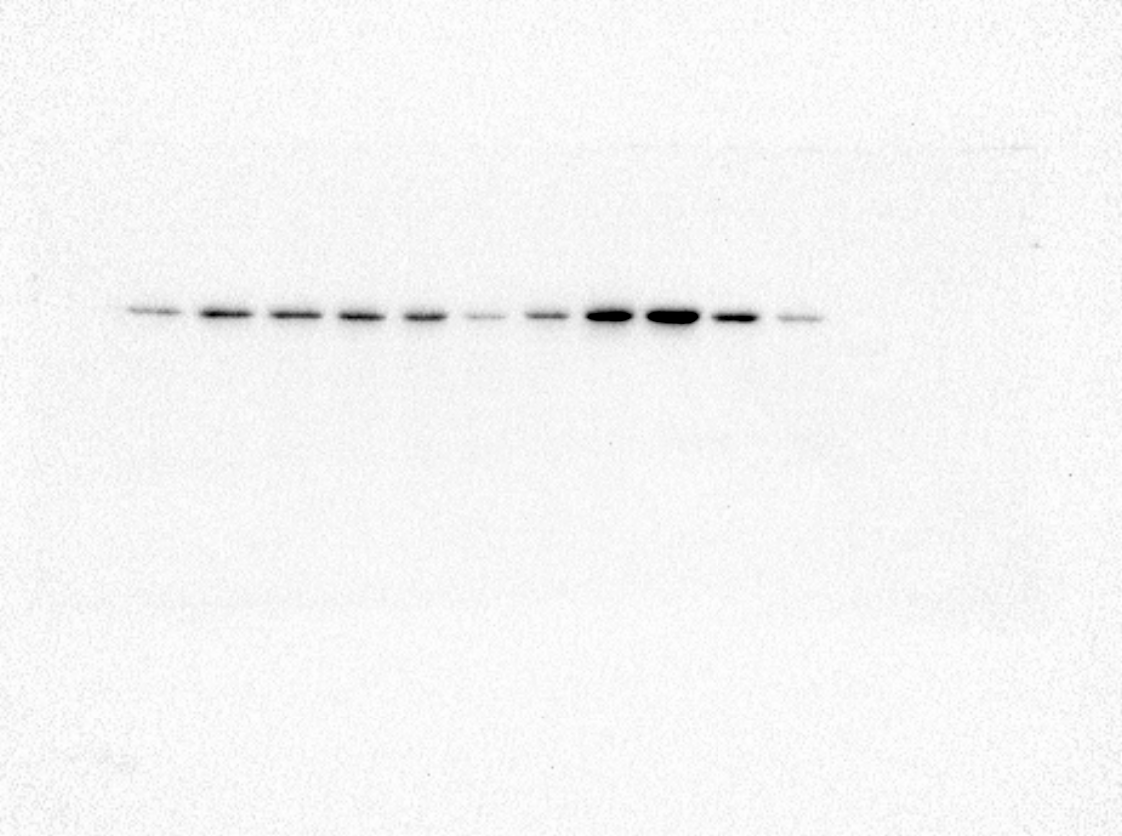

Supplement: Figure 1—figure supplement 1—source data 1. — Figure with the uncropped blots with relevant bands clearly labeled are provided. [file elife-85872-fig1-figsupp1-data1.zip › Figure 1- Figure Supplement 1 - Source Data 3/a-pSRC.tif]

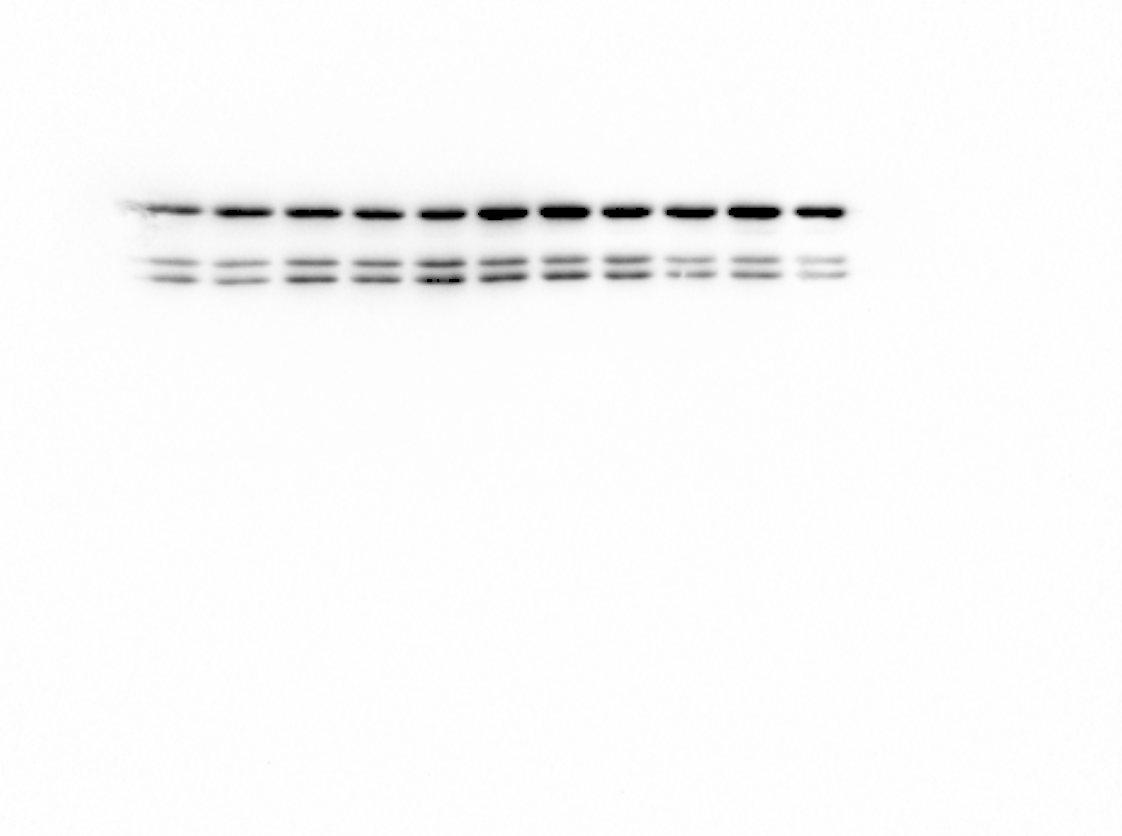

Supplement: Figure 1—figure supplement 1—source data 1. — Figure with the uncropped blots with relevant bands clearly labeled are provided. [file elife-85872-fig1-figsupp1-data1.zip › Figure 1- Figure Supplement 1 - Source Data 3/a-SRC.tif]

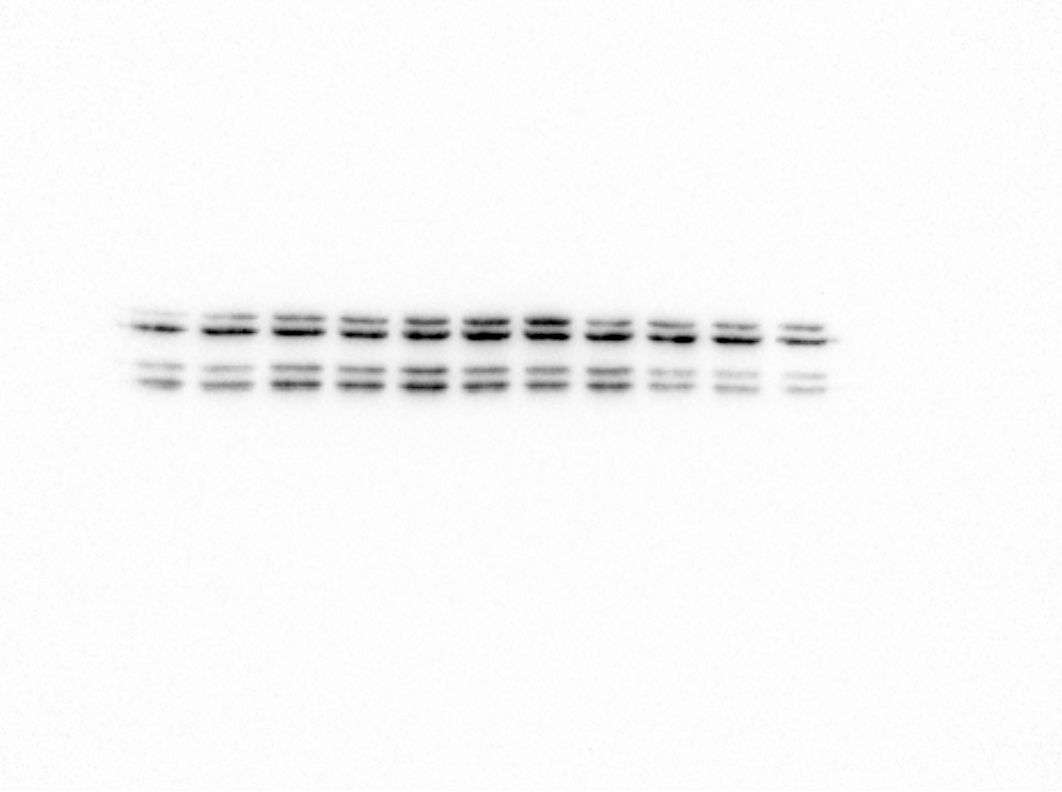

Supplement: Figure 1—figure supplement 1—source data 1. — Figure with the uncropped blots with relevant bands clearly labeled are provided. [file elife-85872-fig1-figsupp1-data1.zip › Figure 1- Figure Supplement 1 - Source Data 3/a-tubulin.tif]

pSrc

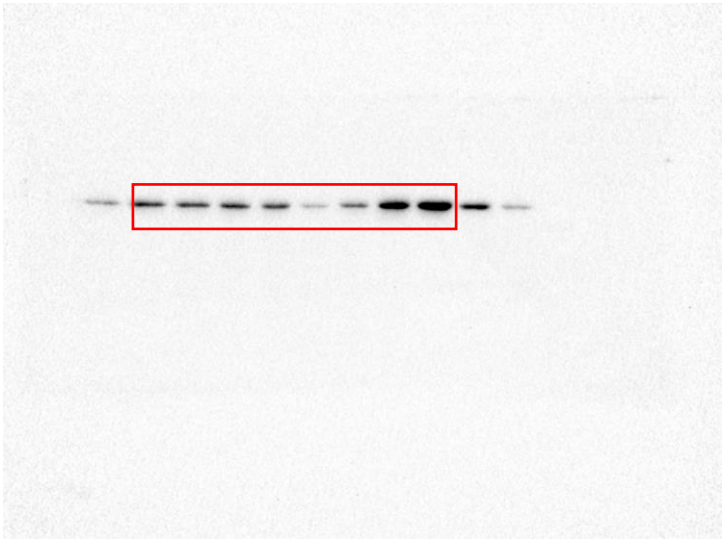

Src

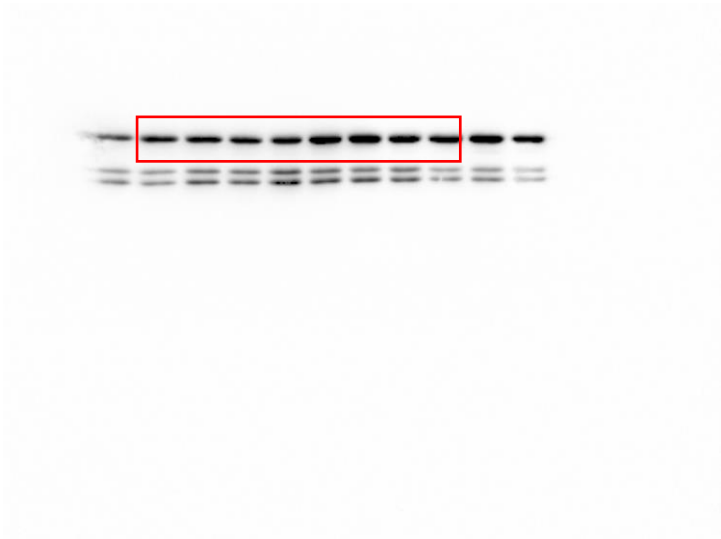

Tubulin

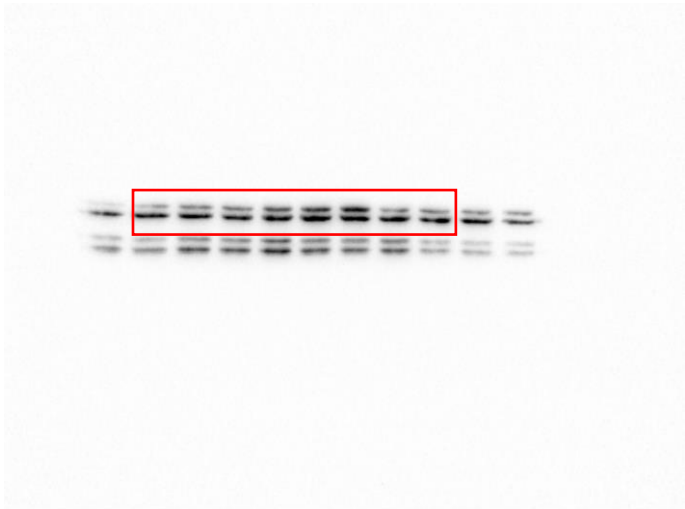

Supplement: Figure 1—figure supplement 1—source data 1. — Figure with the uncropped blots with relevant bands clearly labeled are provided. [file elife-85872-fig1-figsupp1-data1.zip › Figure 1- Figure Supplement 1 - Source Data 3/Rossini et al- Figure 1- Supplement figure 1-uncropped.pdf]

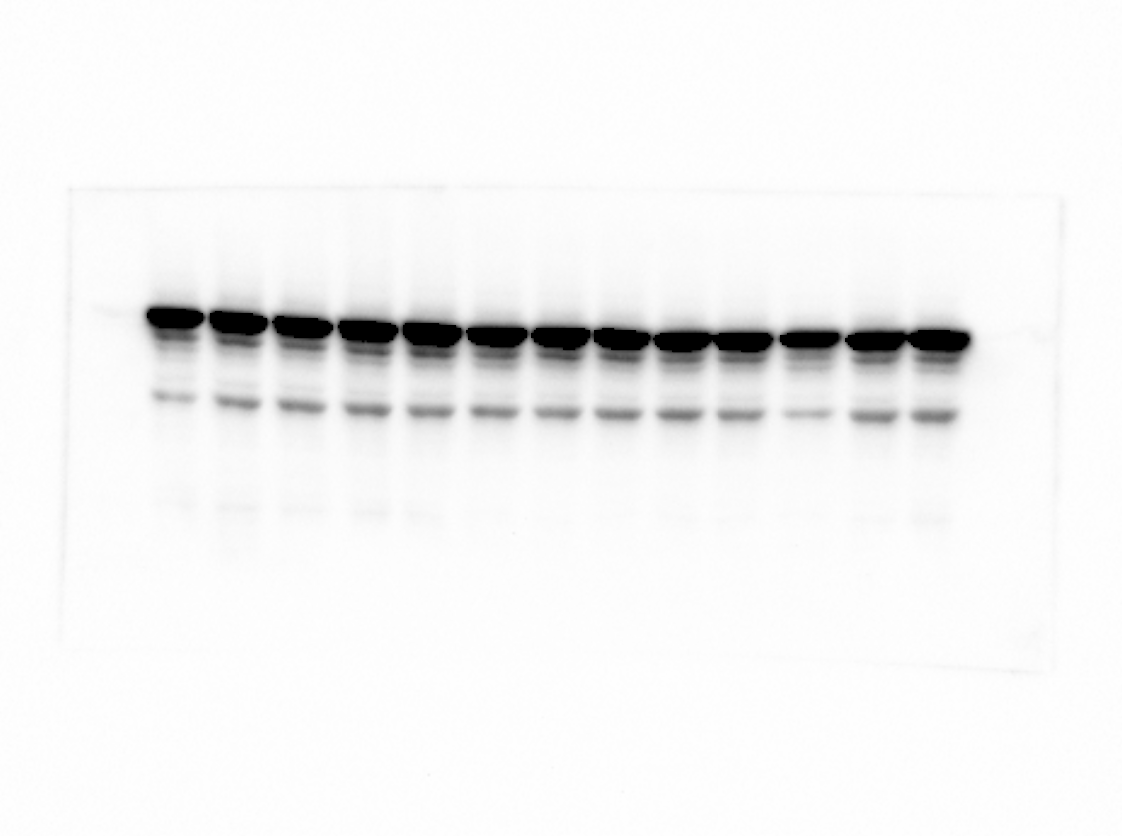

Supplement: Figure 2—source data 1. — Cells were either treated with spermidine (100 µM) or left untreated. Figure with the uncropped blots with relevant bands clearly labeled are provided. [file elife-85872-fig2-data1.zip › Figure 2-source data 4/a-ACTIN.tif]

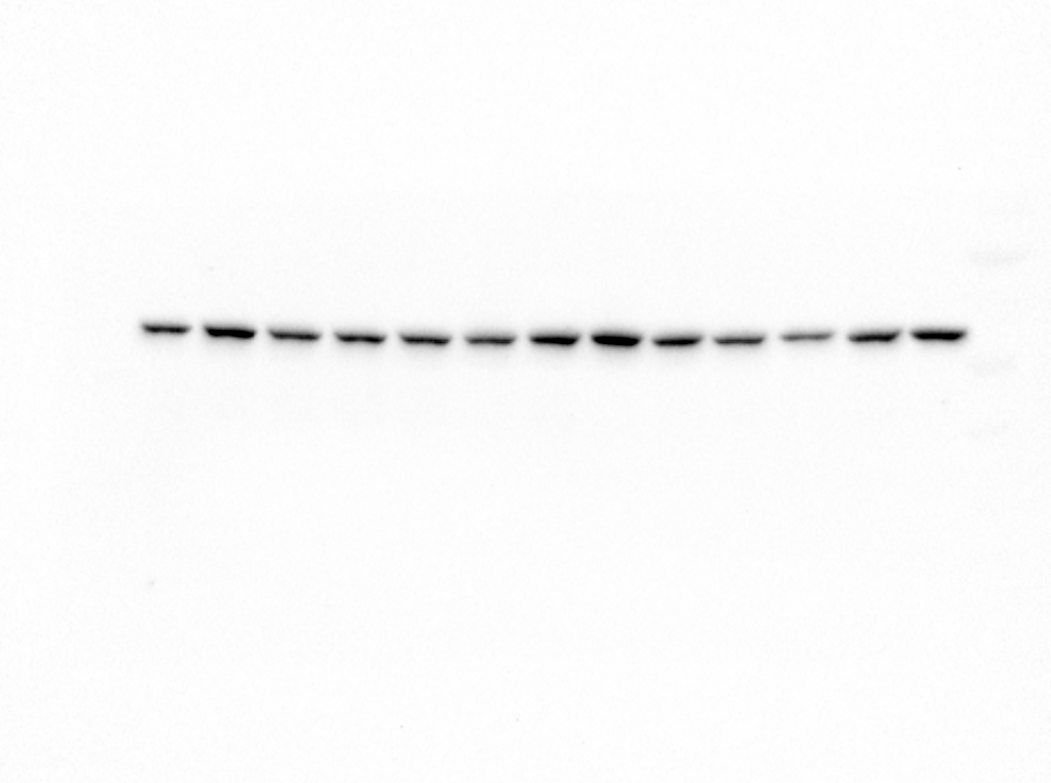

Supplement: Figure 2—source data 1. — Cells were either treated with spermidine (100 µM) or left untreated. Figure with the uncropped blots with relevant bands clearly labeled are provided. [file elife-85872-fig2-data1.zip › Figure 2-source data 4/a-pSrc.tif]

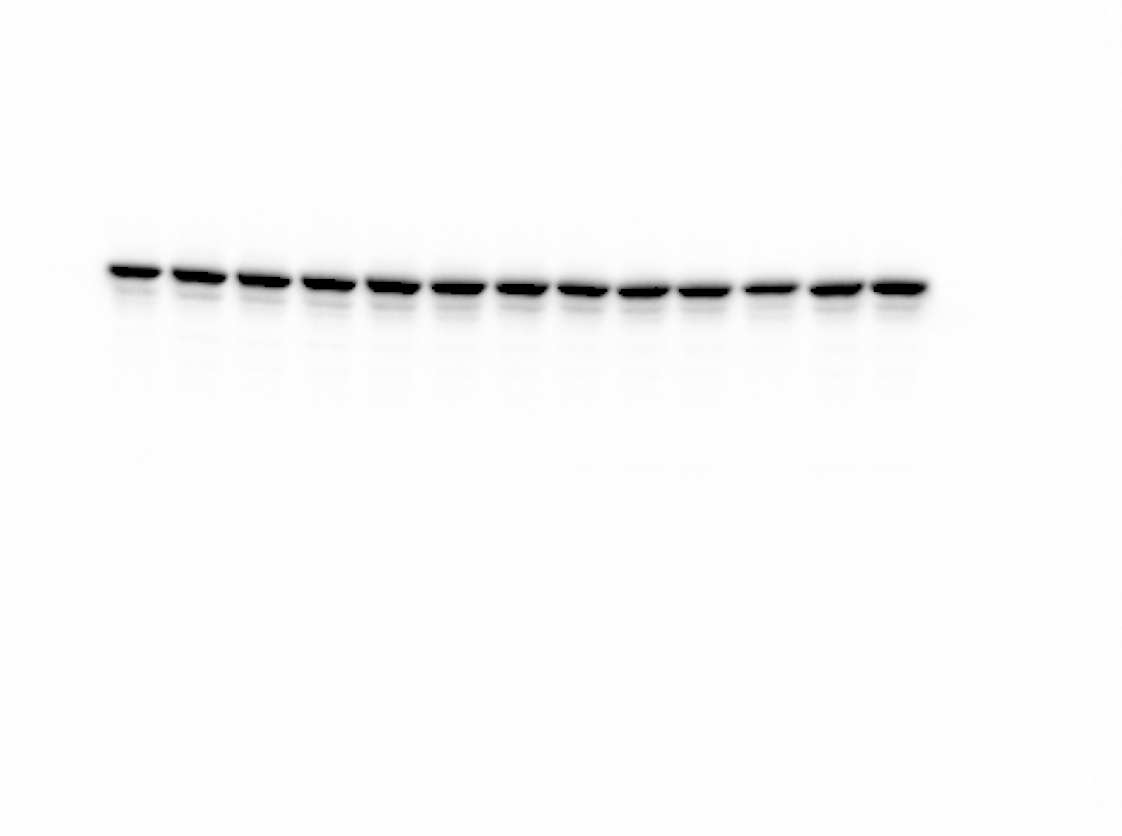

Supplement: Figure 2—source data 1. — Cells were either treated with spermidine (100 µM) or left untreated. Figure with the uncropped blots with relevant bands clearly labeled are provided. [file elife-85872-fig2-data1.zip › Figure 2-source data 4/a-Src.tif]

Rossini et al. Raw Figure 2E (revised)

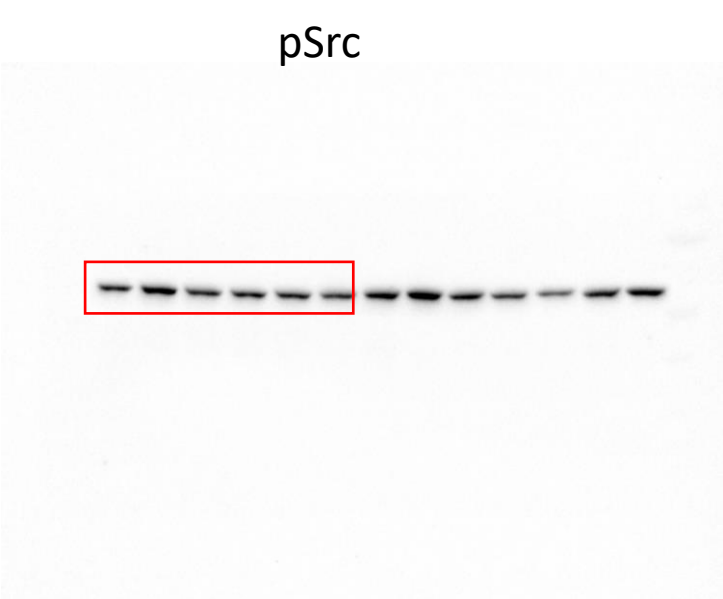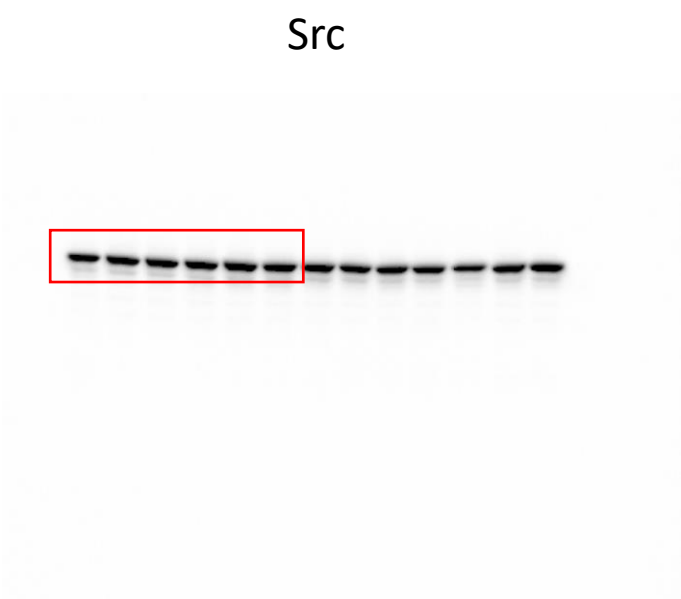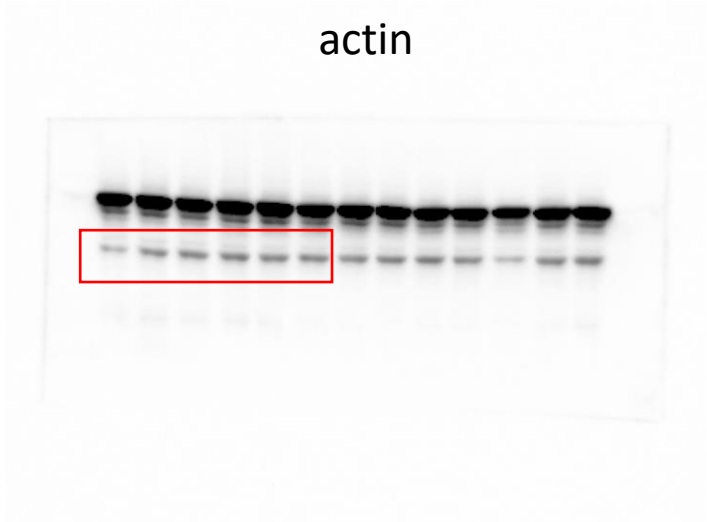

Supplement: Figure 2—source data 1. — Cells were either treated with spermidine (100 µM) or left untreated. Figure with the uncropped blots with relevant bands clearly labeled are provided. [file elife-85872-fig2-data1.zip › Figure 2-source data 4/Rossini et al. Figure 2E_ uncropped.pdf]

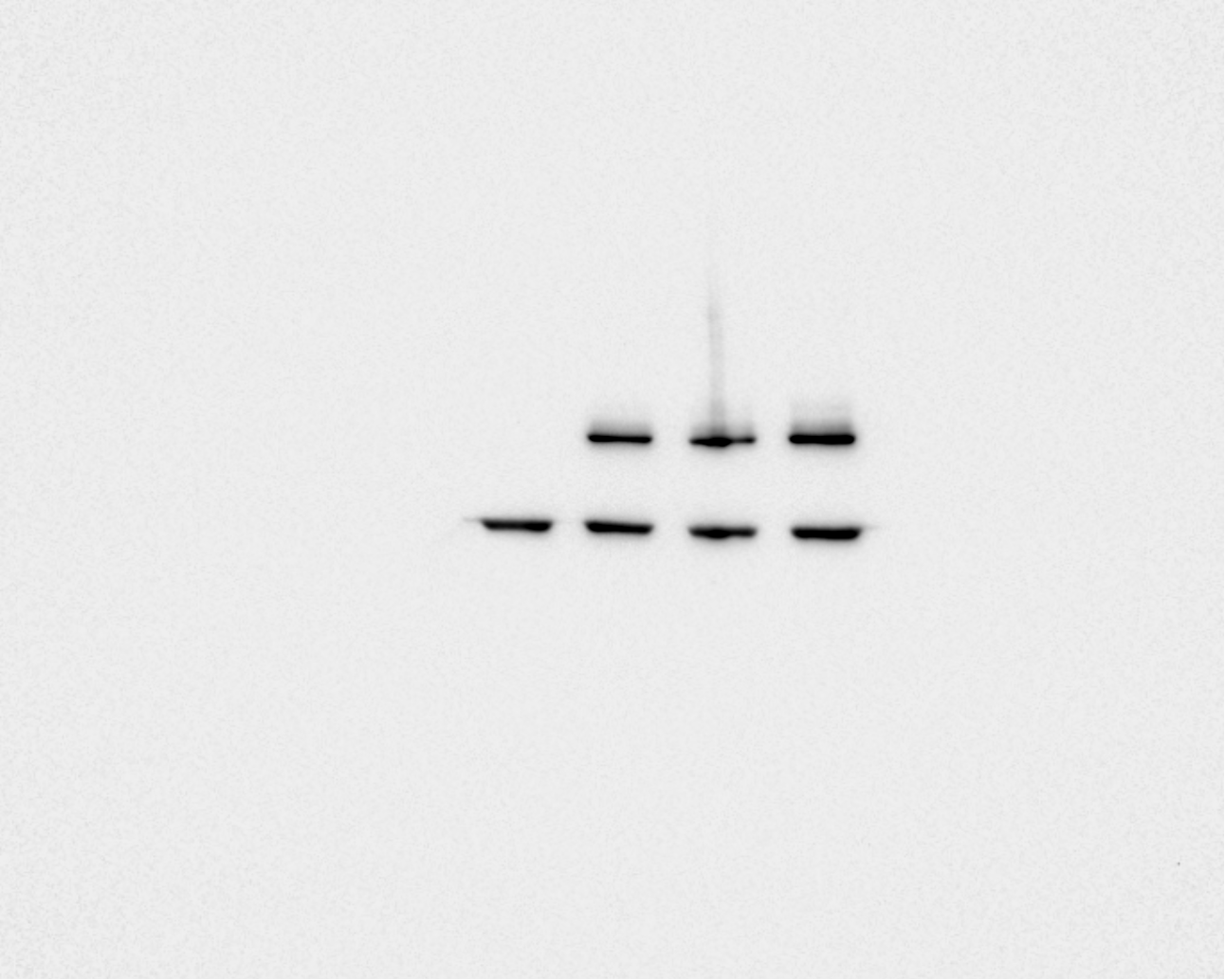

Supplement: Figure 2—figure supplement 2—source data 1. — SYF cells transfected with empty vector (SYF) were used as control. Figure with the uncropped blots with relevant bands clearly labeled are provided. [file elife-85872-fig2-figsupp2-data1.zip › Figure 2 - Figure Supplement 2 - Source Data 5/a-actin.tif]

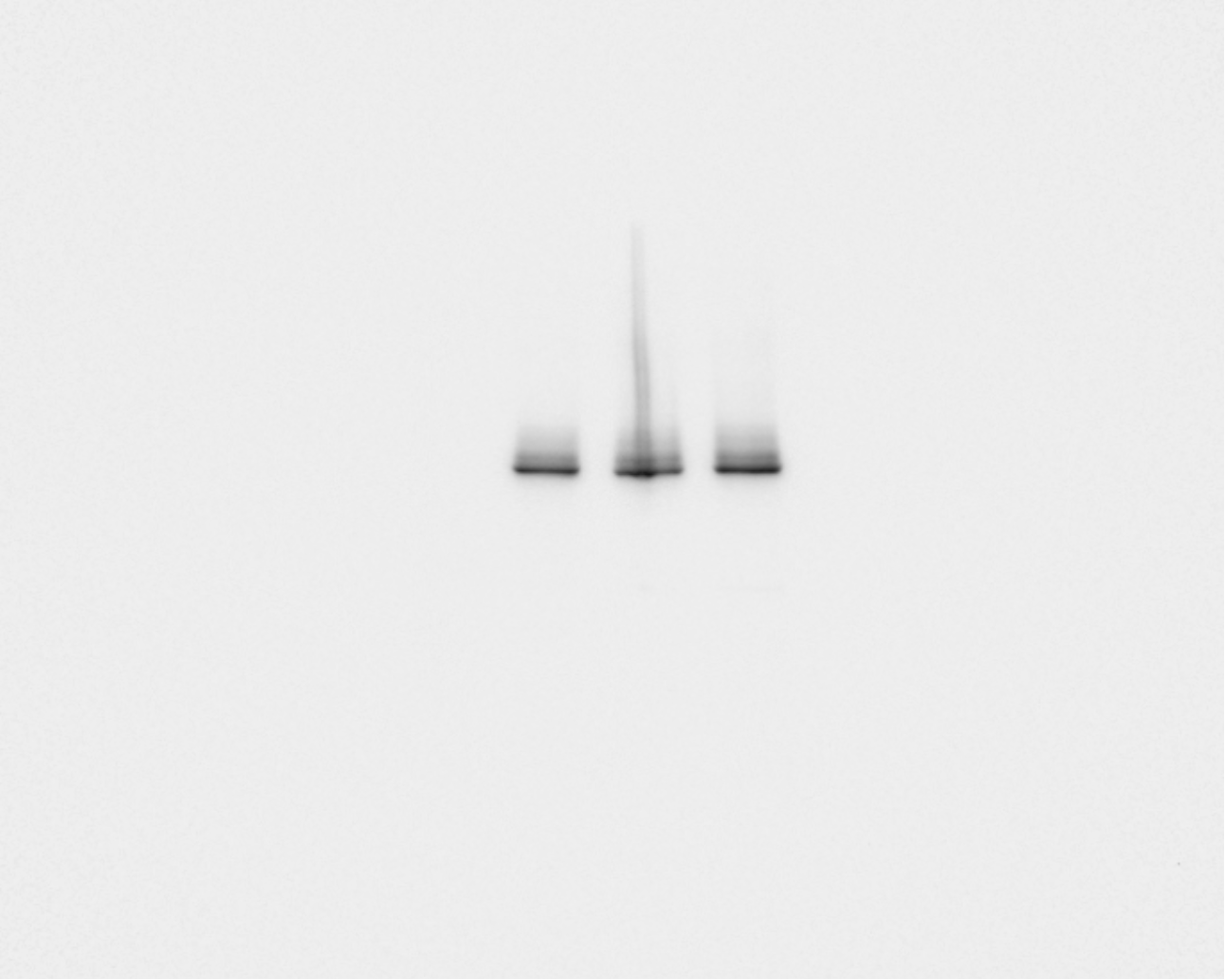

Supplement: Figure 2—figure supplement 2—source data 1. — SYF cells transfected with empty vector (SYF) were used as control. Figure with the uncropped blots with relevant bands clearly labeled are provided. [file elife-85872-fig2-figsupp2-data1.zip › Figure 2 - Figure Supplement 2 - Source Data 5/a-pSRC.tif]

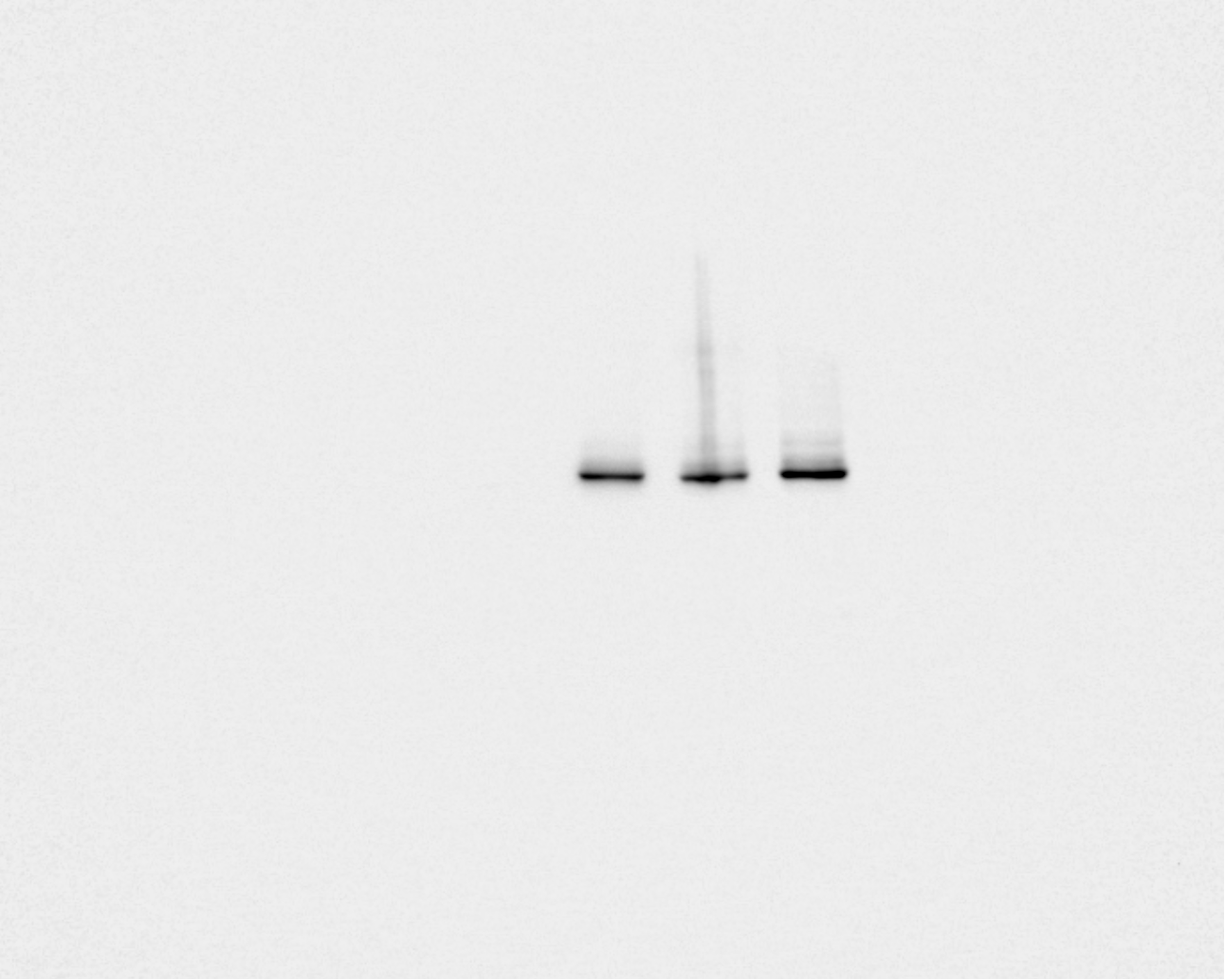

Supplement: Figure 2—figure supplement 2—source data 1. — SYF cells transfected with empty vector (SYF) were used as control. Figure with the uncropped blots with relevant bands clearly labeled are provided. [file elife-85872-fig2-figsupp2-data1.zip › Figure 2 - Figure Supplement 2 - Source Data 5/a-SRC.tif]

pSrc

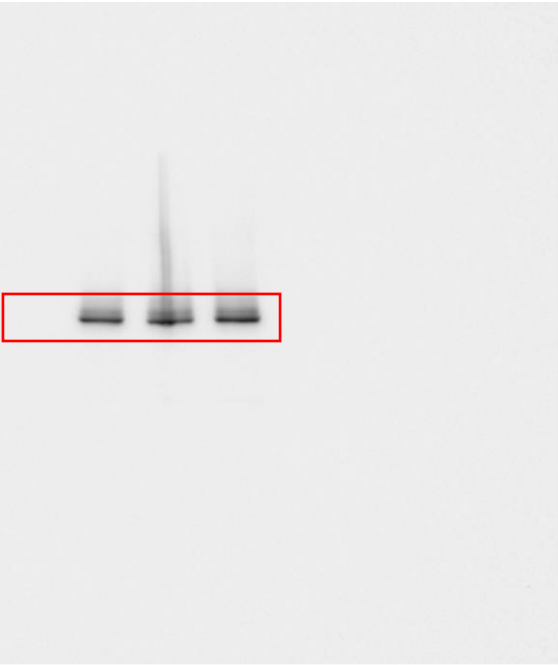

Src

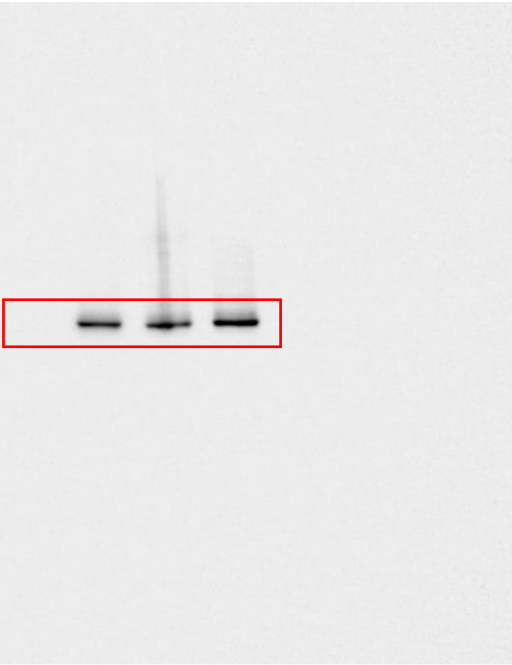

Actin

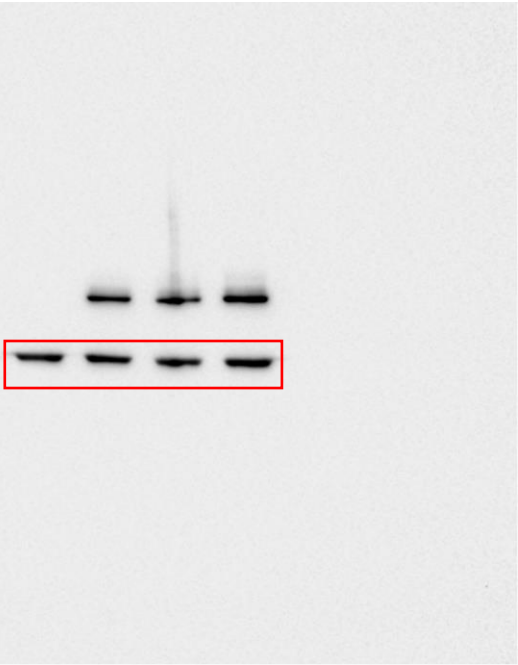

Supplement: Figure 2—figure supplement 2—source data 1. — SYF cells transfected with empty vector (SYF) were used as control. Figure with the uncropped blots with relevant bands clearly labeled are provided. [file elife-85872-fig2-figsupp2-data1.zip › Figure 2 - Figure Supplement 2 - Source Data 5/Rossini et al. Figure 2-Supplement figure 2a_uncropped.pdf]

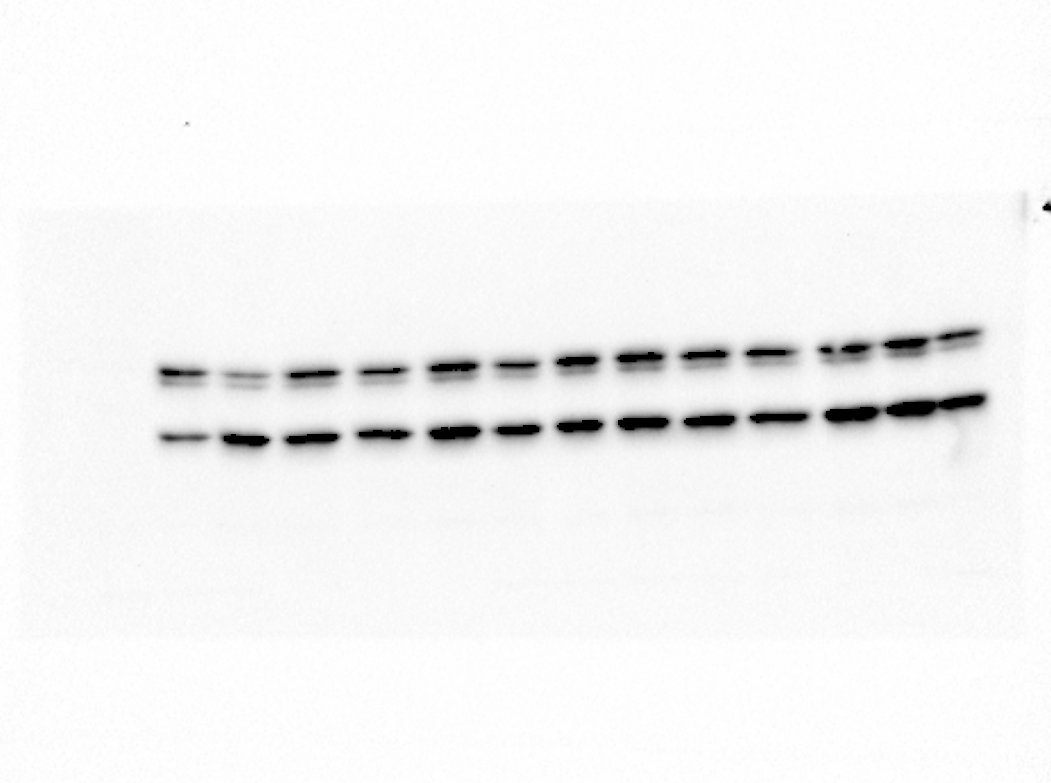

Supplement: Figure 2—figure supplement 2—source data 2. — Figure with the uncropped blots with relevant bands clearly labeled are provided. [file elife-85872-fig2-figsupp2-data2.zip › Figure 2 - Figure Supplement 2 - Source Data 6/a-Actin.tif]

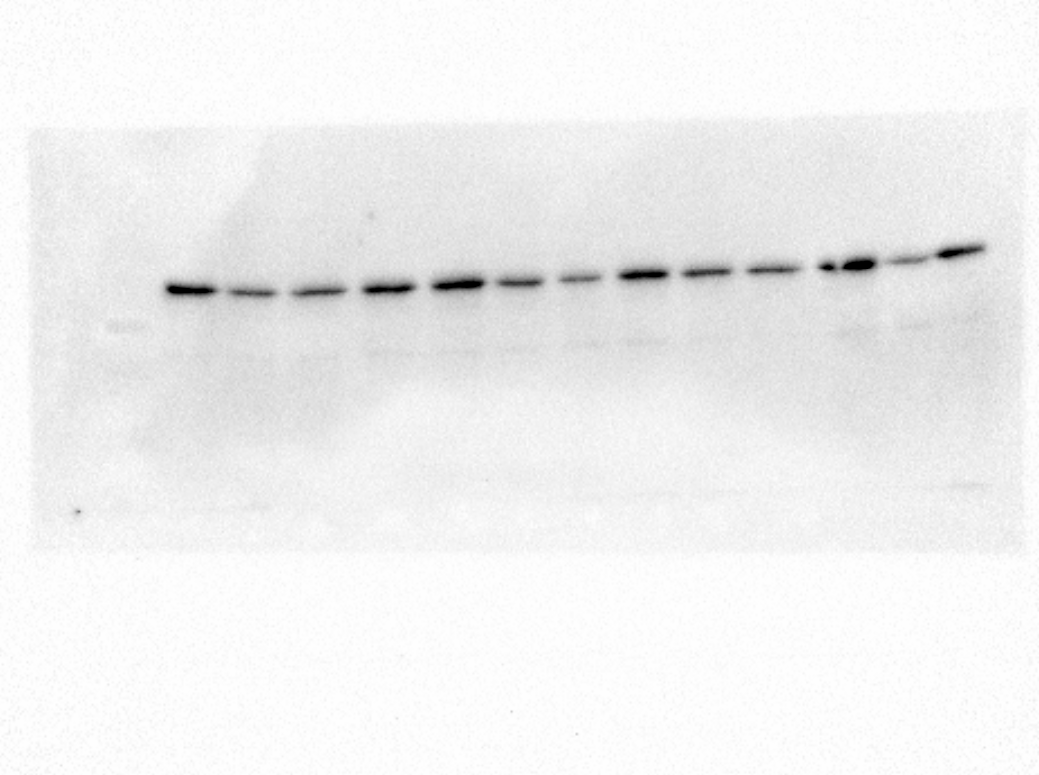

Supplement: Figure 2—figure supplement 2—source data 2. — Figure with the uncropped blots with relevant bands clearly labeled are provided. [file elife-85872-fig2-figsupp2-data2.zip › Figure 2 - Figure Supplement 2 - Source Data 6/a-pSRC.tif]

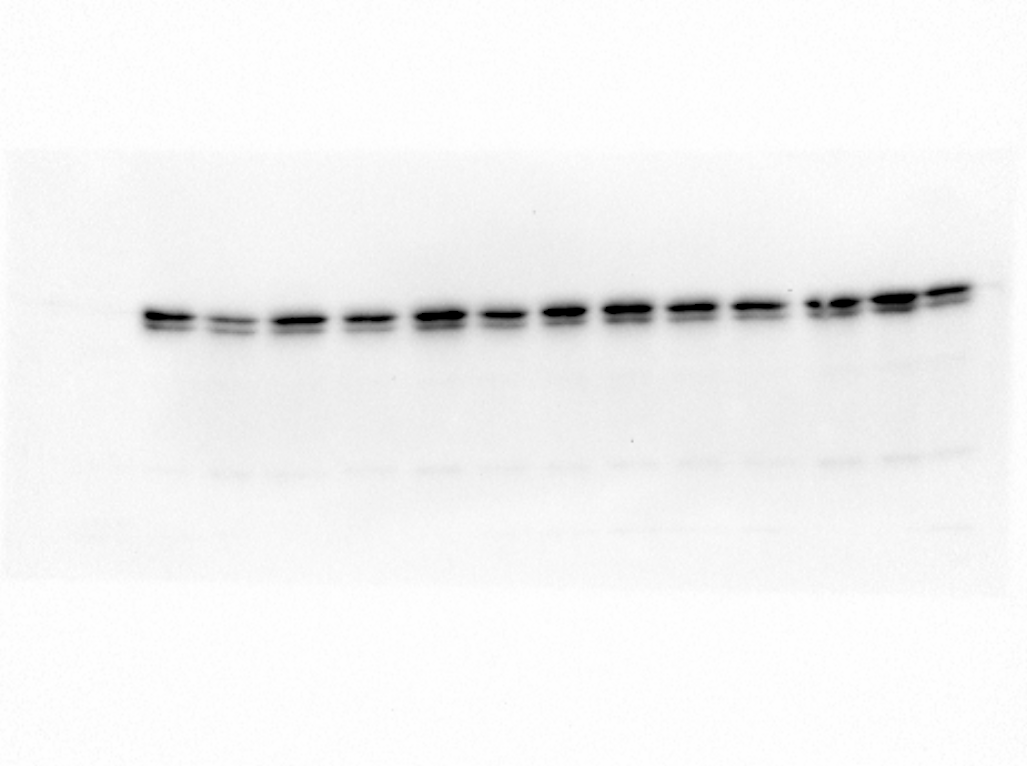

Supplement: Figure 2—figure supplement 2—source data 2. — Figure with the uncropped blots with relevant bands clearly labeled are provided. [file elife-85872-fig2-figsupp2-data2.zip › Figure 2 - Figure Supplement 2 - Source Data 6/a-SRC.tif]

pSrc

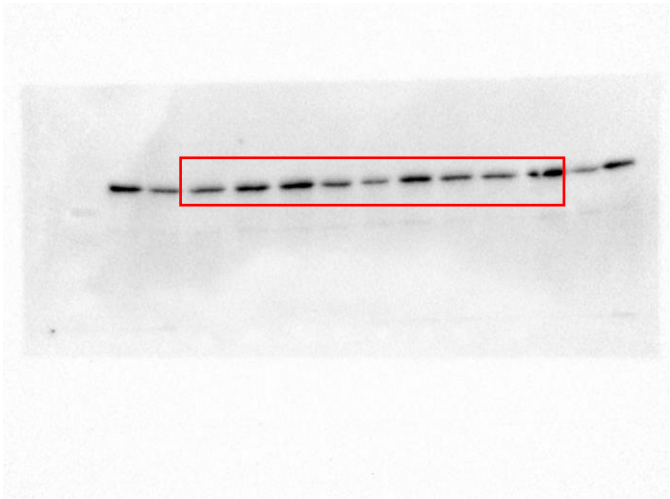

Src

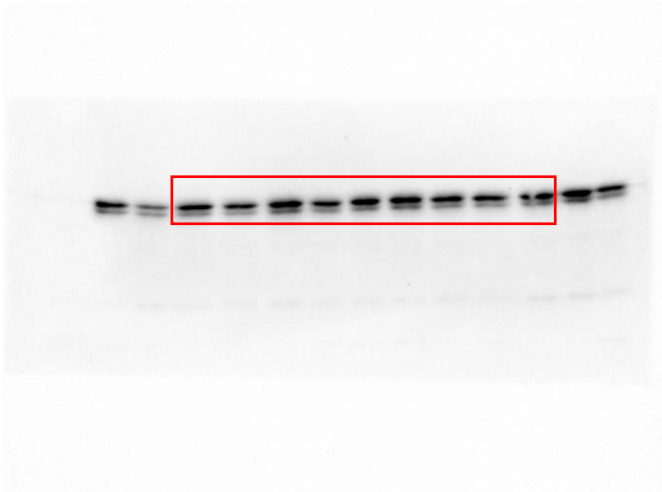

actin

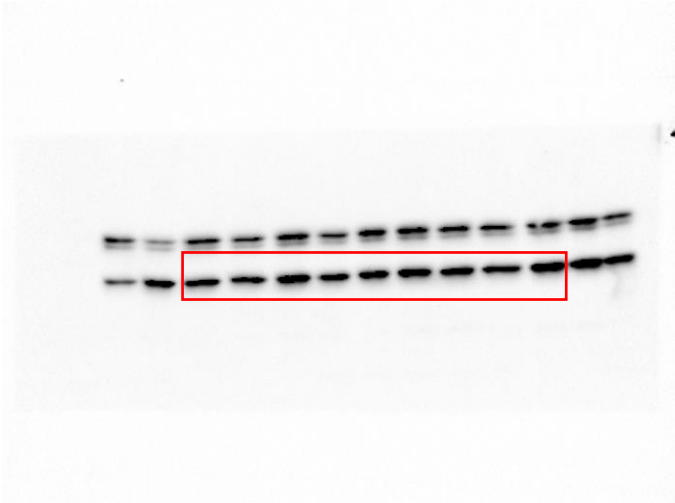

Supplement: Figure 2—figure supplement 2—source data 2. — Figure with the uncropped blots with relevant bands clearly labeled are provided. [file elife-85872-fig2-figsupp2-data2.zip › Figure 2 - Figure Supplement 2 - Source Data 6/Rossini et al. Figure 2-Supplement figure 2b_uncropped.pdf]

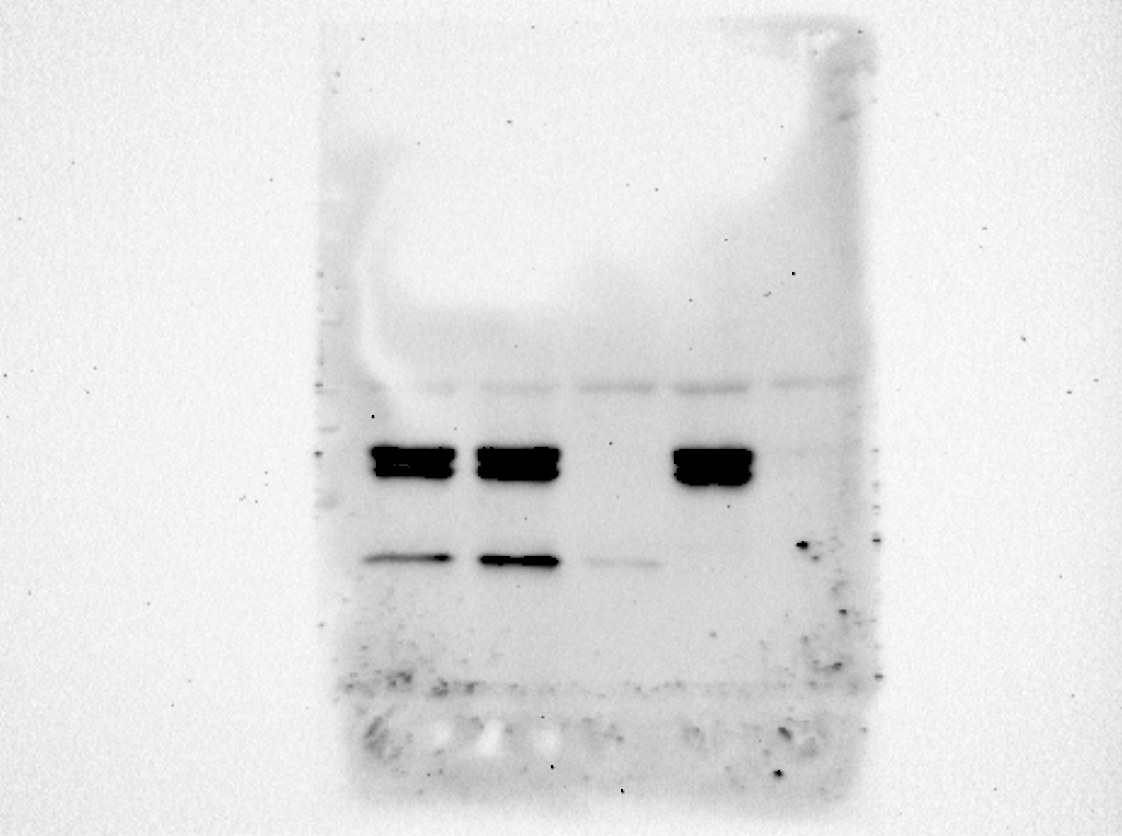

Supplement: Figure 3—source data 1. — Whole-cell lysates (PRE-IP) was used as control of protein expression of IDO1, Src, and β-tubulin. SYF cells reconstituted with vectors coding for Src and IDO1 and then treated with spermidine (100 μM) for 60 min as well as cells transfected with vectors coding for either Src or IDO1 were used for the experiments. The negative control (i.e., the sample expressing both IDO1 and Src, but not immunoprecipitated with the antibody) is included. Figure with the uncropped blots with relevant bands clearly labeled are provided. [file elife-85872-fig3-data1.zip › Figure 3-source data 7/IP apTyr_a-IDO1.tif]

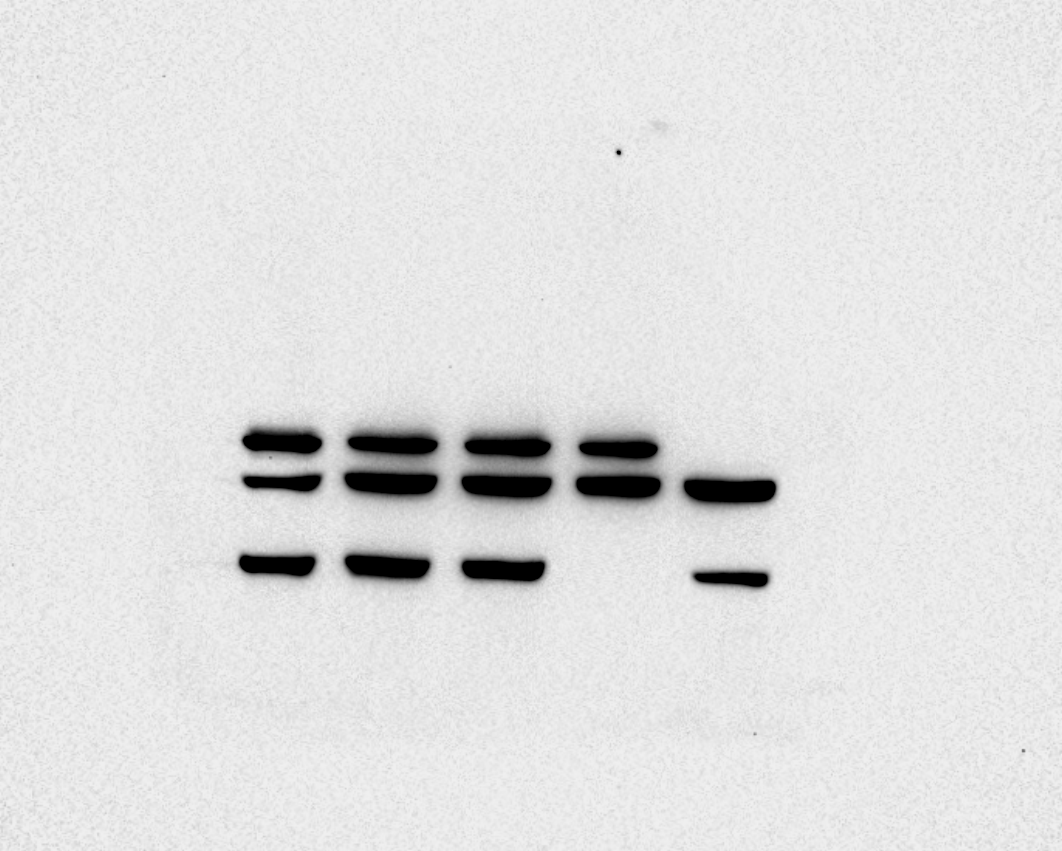

Supplement: Figure 3—source data 1. — Whole-cell lysates (PRE-IP) was used as control of protein expression of IDO1, Src, and β-tubulin. SYF cells reconstituted with vectors coding for Src and IDO1 and then treated with spermidine (100 μM) for 60 min as well as cells transfected with vectors coding for either Src or IDO1 were used for the experiments. The negative control (i.e., the sample expressing both IDO1 and Src, but not immunoprecipitated with the antibody) is included. Figure with the uncropped blots with relevant bands clearly labeled are provided. [file elife-85872-fig3-data1.zip › Figure 3-source data 7/PRE IP_a-btubulin.tif]

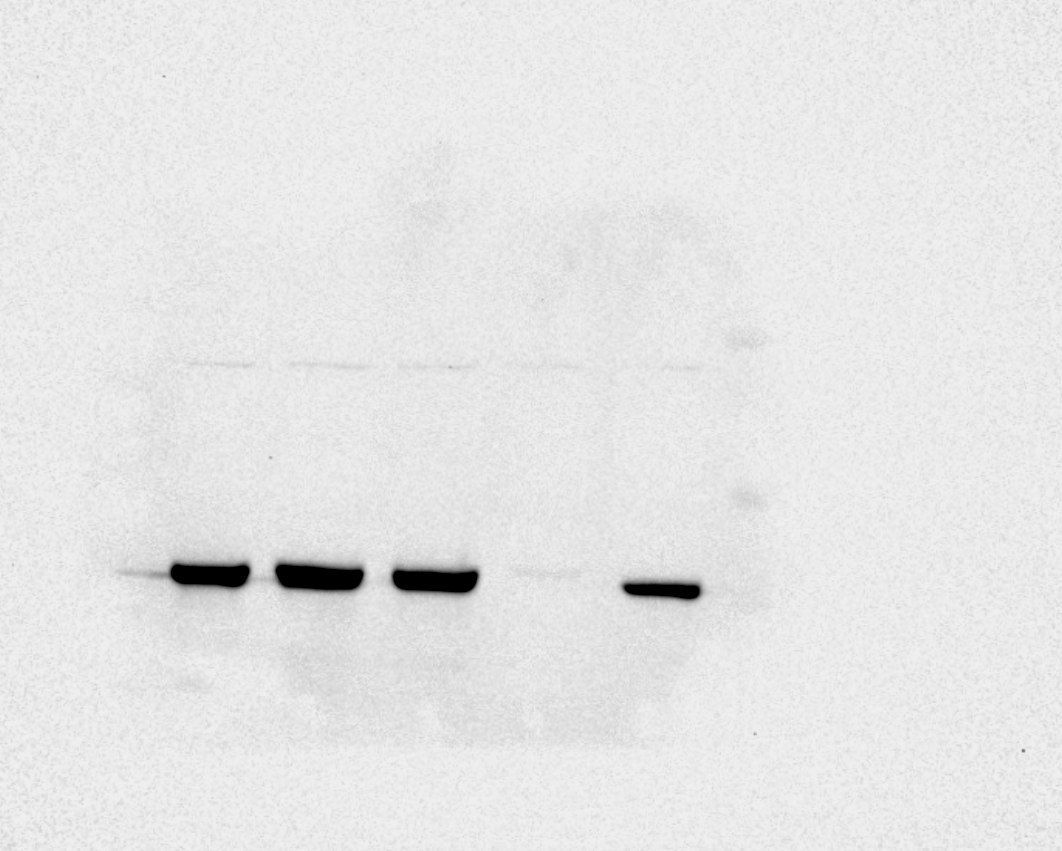

Supplement: Figure 3—source data 1. — Whole-cell lysates (PRE-IP) was used as control of protein expression of IDO1, Src, and β-tubulin. SYF cells reconstituted with vectors coding for Src and IDO1 and then treated with spermidine (100 μM) for 60 min as well as cells transfected with vectors coding for either Src or IDO1 were used for the experiments. The negative control (i.e., the sample expressing both IDO1 and Src, but not immunoprecipitated with the antibody) is included. Figure with the uncropped blots with relevant bands clearly labeled are provided. [file elife-85872-fig3-data1.zip › Figure 3-source data 7/PRE IP_a-IDO1.tif]

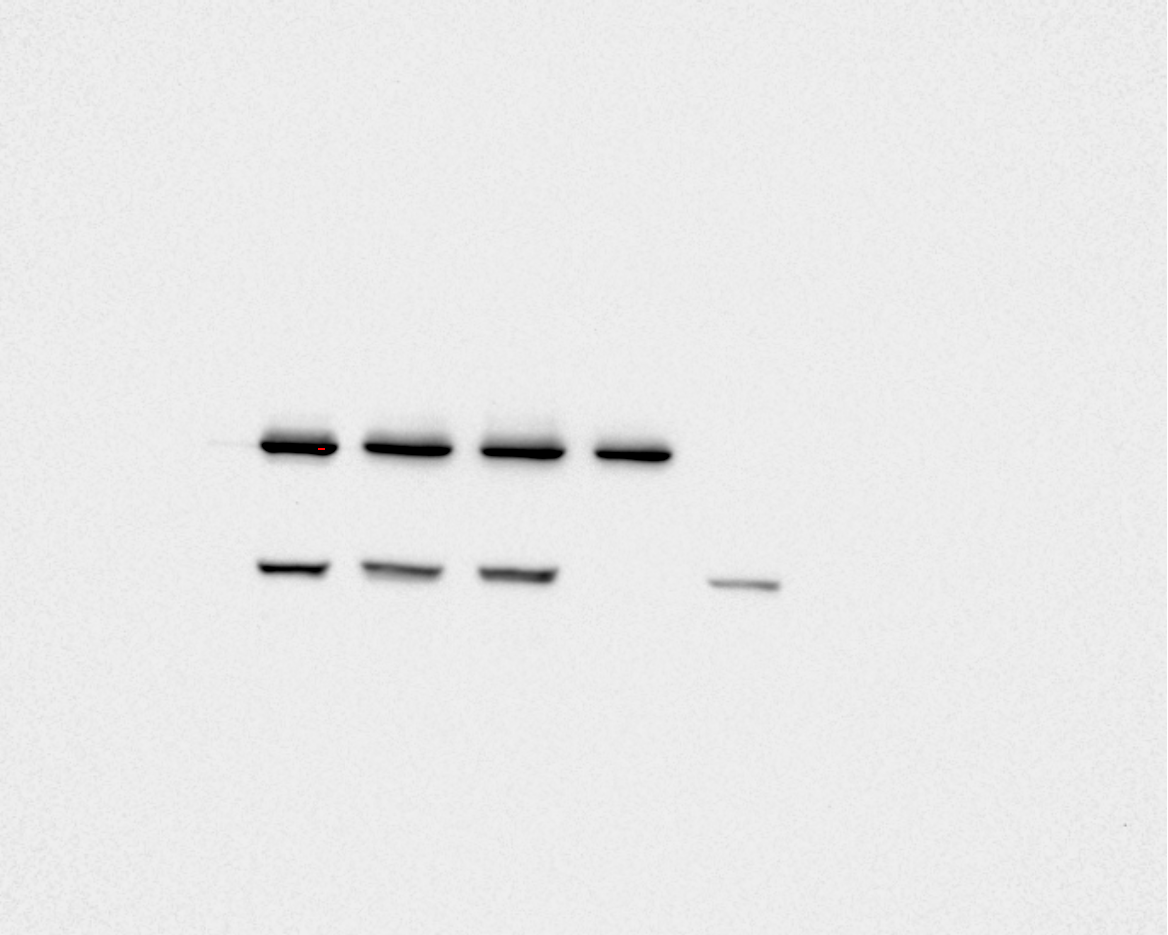

Supplement: Figure 3—source data 1. — Whole-cell lysates (PRE-IP) was used as control of protein expression of IDO1, Src, and β-tubulin. SYF cells reconstituted with vectors coding for Src and IDO1 and then treated with spermidine (100 μM) for 60 min as well as cells transfected with vectors coding for either Src or IDO1 were used for the experiments. The negative control (i.e., the sample expressing both IDO1 and Src, but not immunoprecipitated with the antibody) is included. Figure with the uncropped blots with relevant bands clearly labeled are provided. [file elife-85872-fig3-data1.zip › Figure 3-source data 7/PRE IP_a-Src.tif]

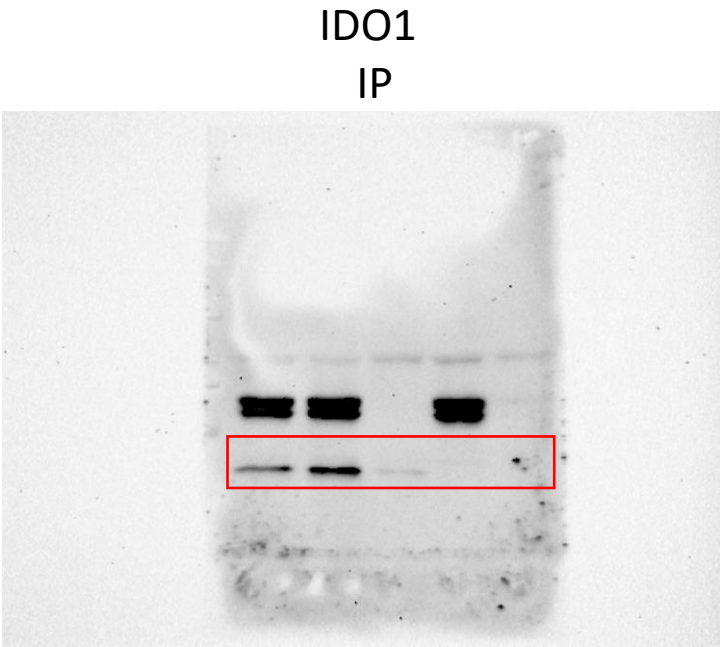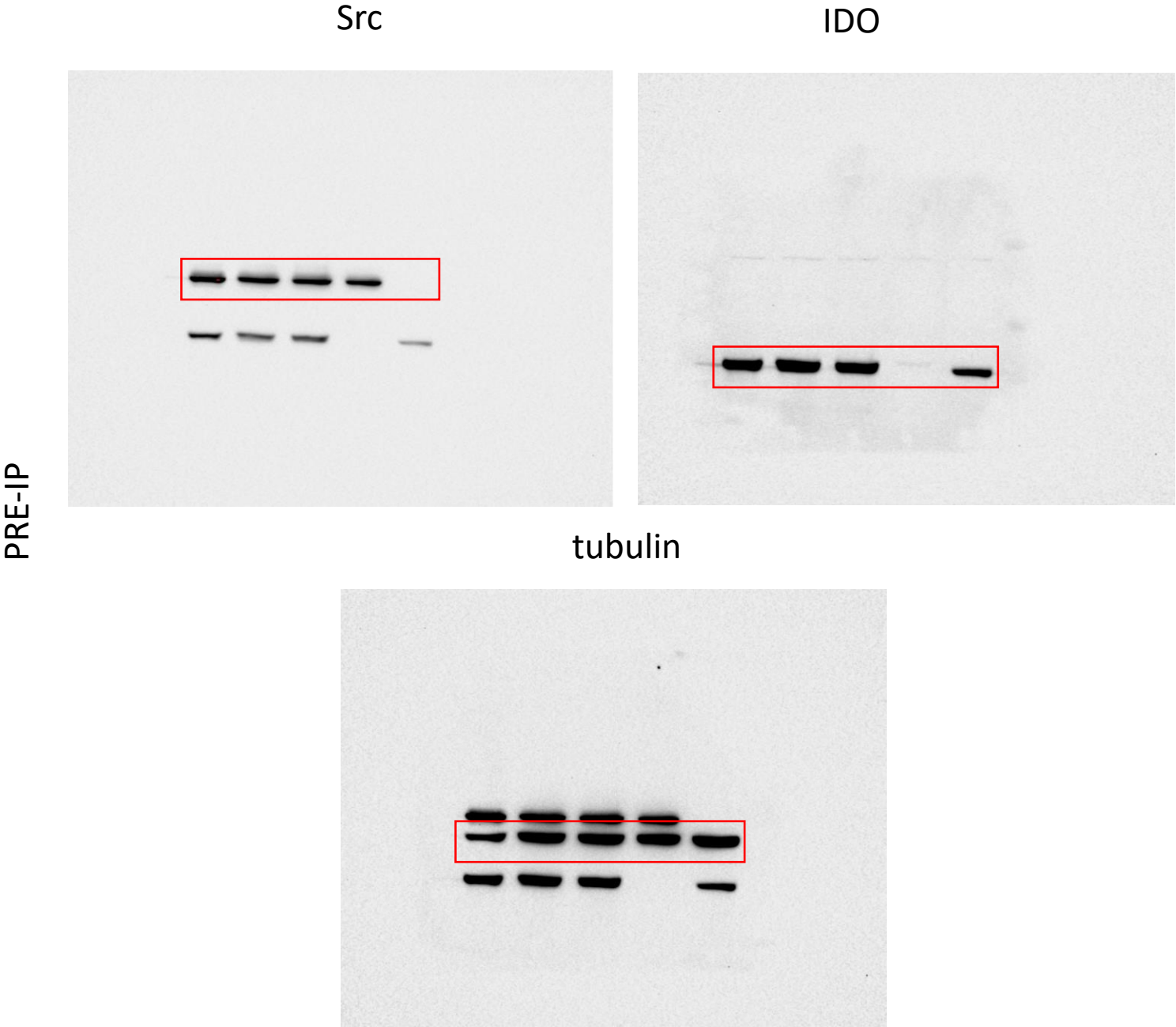

Supplement: Figure 3—source data 1. — Whole-cell lysates (PRE-IP) was used as control of protein expression of IDO1, Src, and β-tubulin. SYF cells reconstituted with vectors coding for Src and IDO1 and then treated with spermidine (100 μM) for 60 min as well as cells transfected with vectors coding for either Src or IDO1 were used for the experiments. The negative control (i.e., the sample expressing both IDO1 and Src, but not immunoprecipitated with the antibody) is included. Figure with the uncropped blots with relevant bands clearly labeled are provided. [file elife-85872-fig3-data1.zip › Figure 3-source data 7/Rossini et al. Figure 3A_uncropped.pdf]

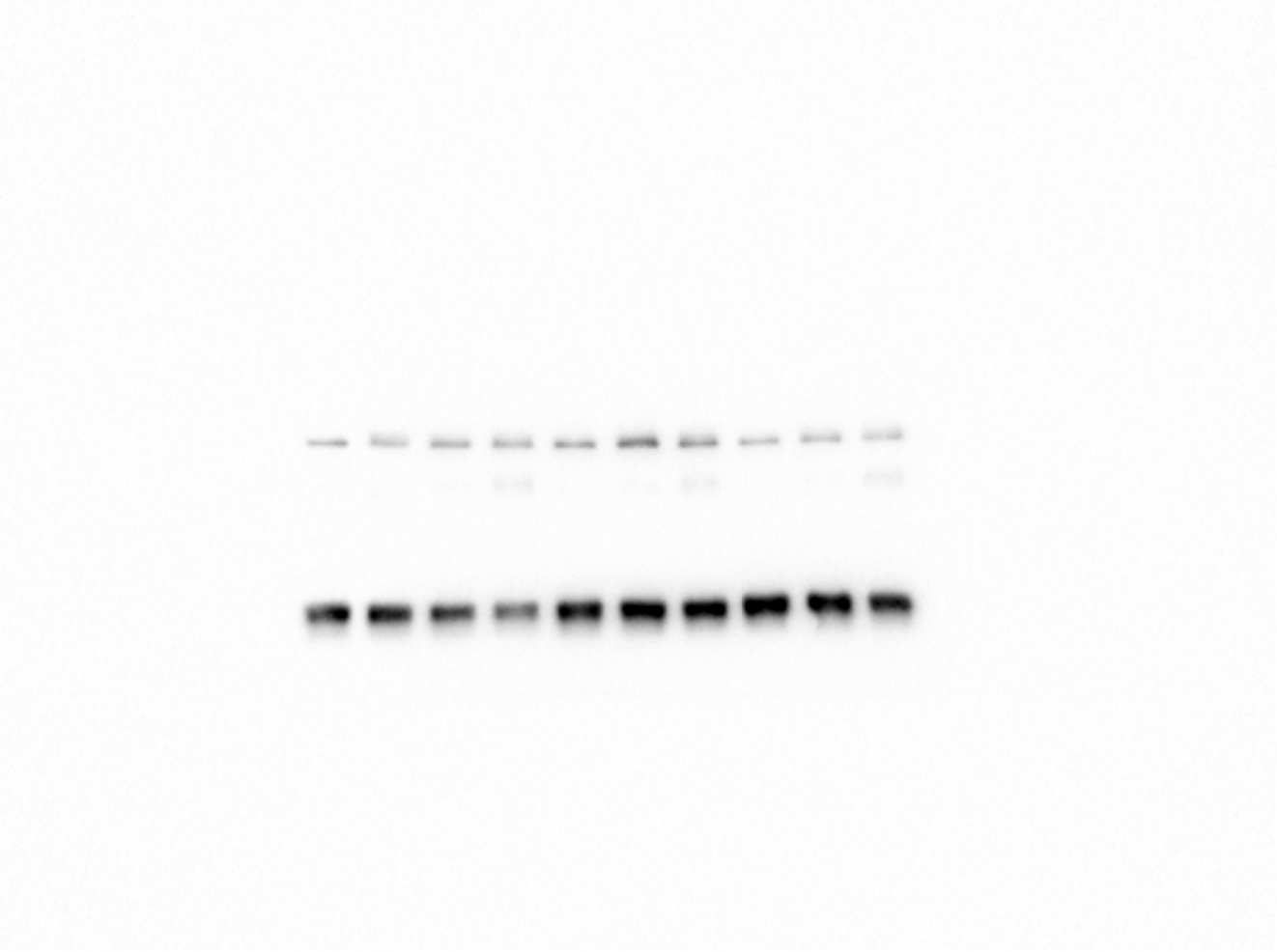

Supplement: Figure 3—source data 2. — The reaction was in either the presence or absence of spermidine. Figure with the uncropped blots with relevant bands clearly labeled are provided. [file elife-85872-fig3-data2.zip › Figure 3-source data 8/a-hIDO.tif]

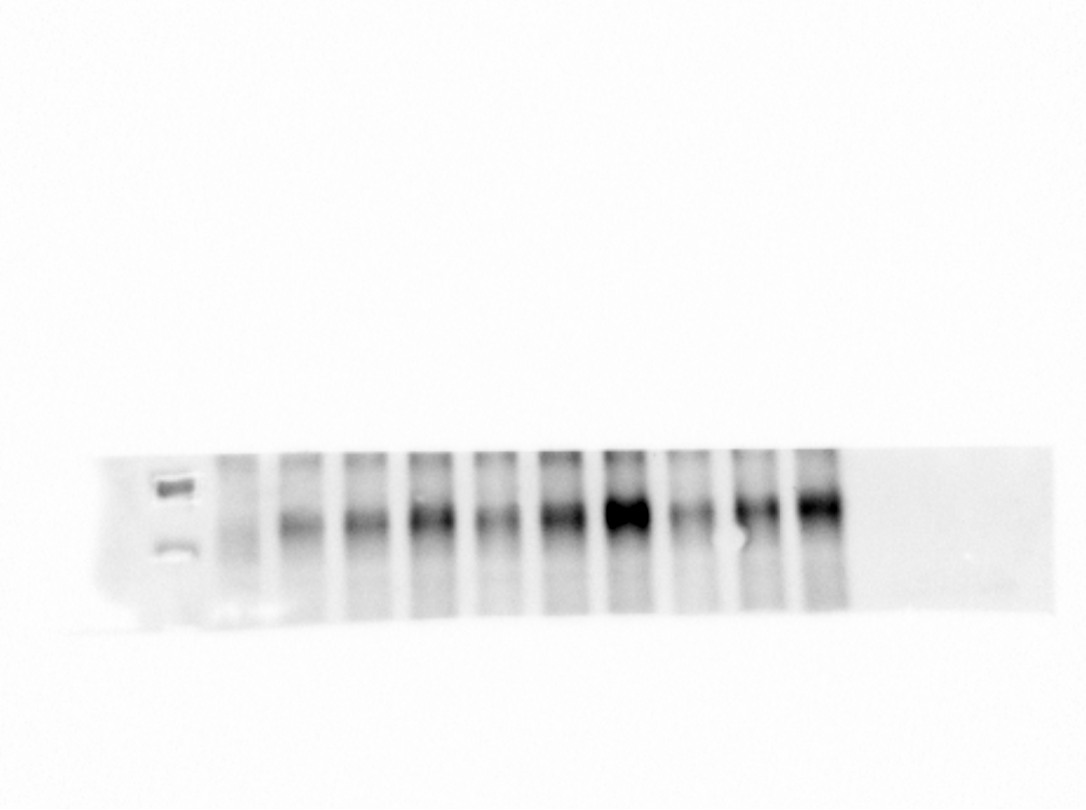

Supplement: Figure 3—source data 2. — The reaction was in either the presence or absence of spermidine. Figure with the uncropped blots with relevant bands clearly labeled are provided. [file elife-85872-fig3-data2.zip › Figure 3-source data 8/a-PTYR.tif]

pTYR

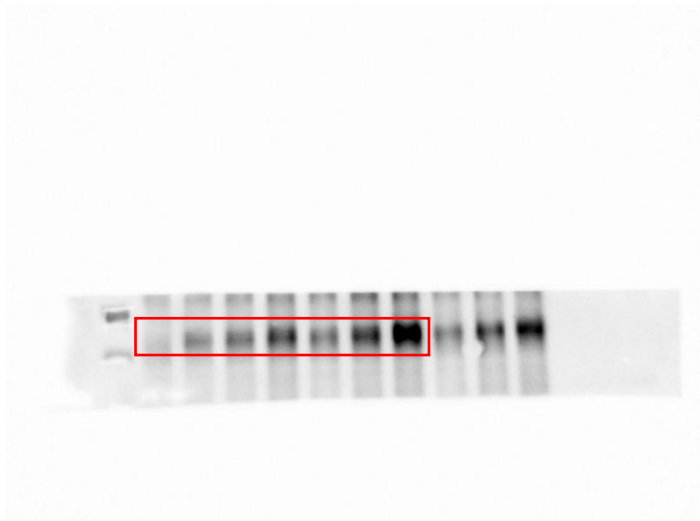

IDO1

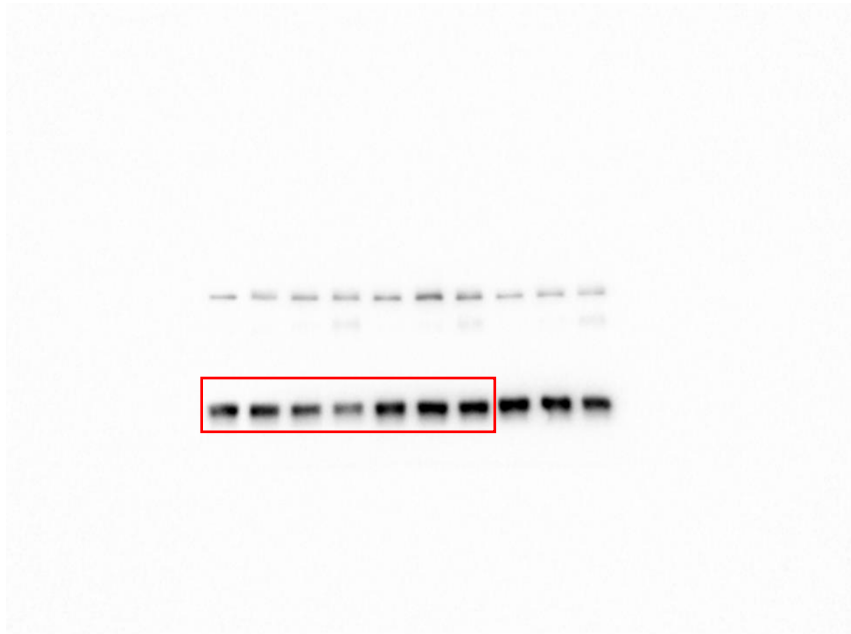

Supplement: Figure 3—source data 2. — The reaction was in either the presence or absence of spermidine. Figure with the uncropped blots with relevant bands clearly labeled are provided. [file elife-85872-fig3-data2.zip › Figure 3-source data 8/Rossini et al. Figure 3B_uncropped.pdf]

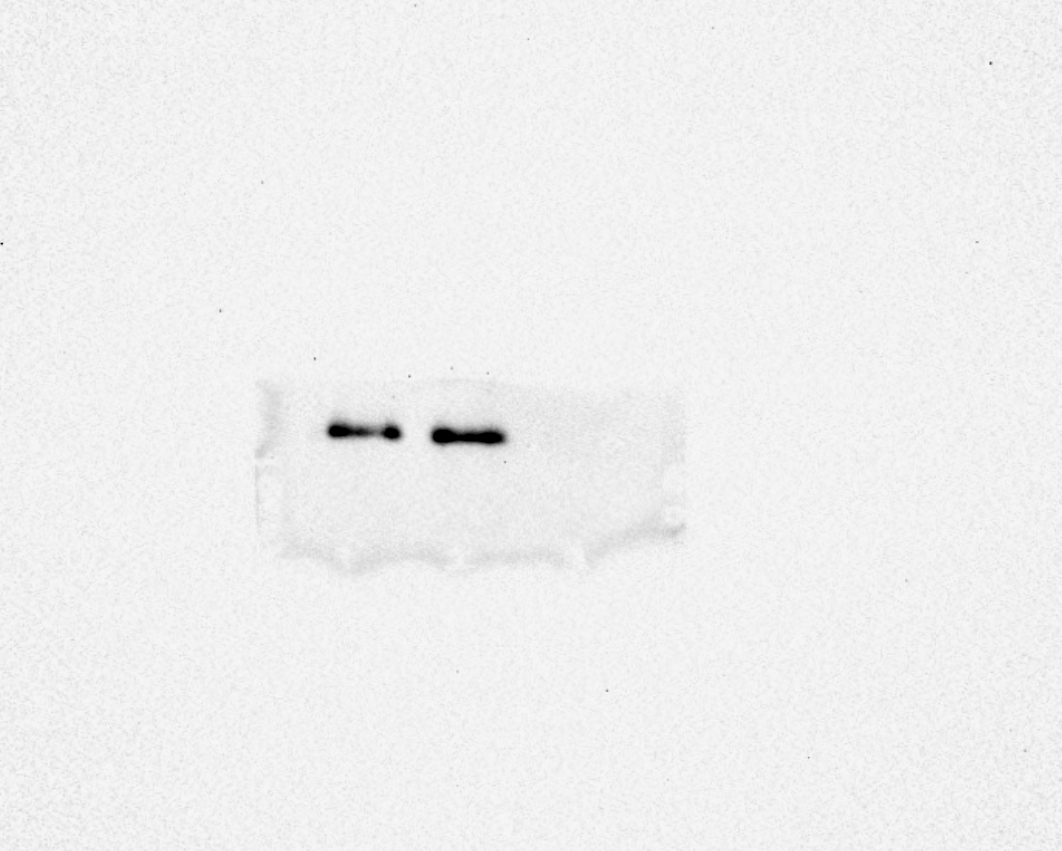

Supplement: Figure 3—source data 3. — Whole-cell lysates (PRE-IP) of MC38 cells was used as control of protein expression of IDO1, Src, and β-actin. The negative control (i.e., sample not immunoprecipitated with the antibody) is included. Figure with the uncropped blots with relevant bands clearly labeled are provided. [file elife-85872-fig3-data3.zip › Figure 3-source data 9/IP_a-IDO1.tif]

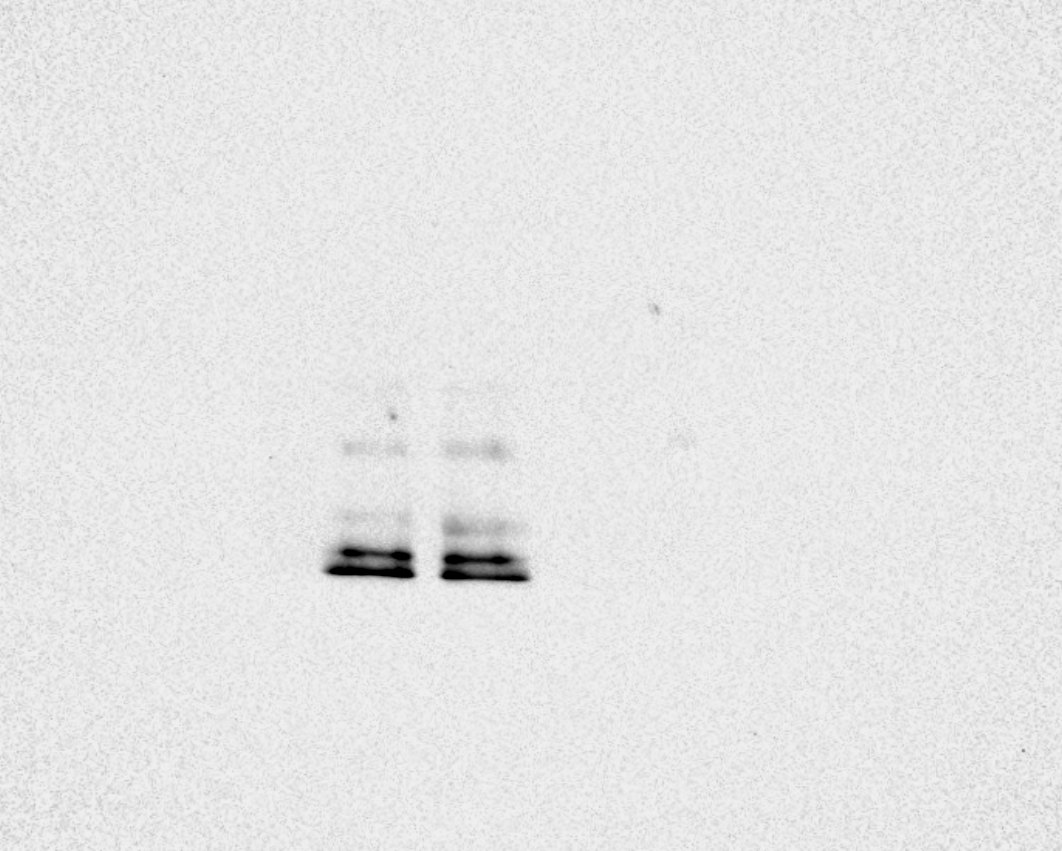

Supplement: Figure 3—source data 3. — Whole-cell lysates (PRE-IP) of MC38 cells was used as control of protein expression of IDO1, Src, and β-actin. The negative control (i.e., sample not immunoprecipitated with the antibody) is included. Figure with the uncropped blots with relevant bands clearly labeled are provided. [file elife-85872-fig3-data3.zip › Figure 3-source data 9/IP_a-Src.tif]

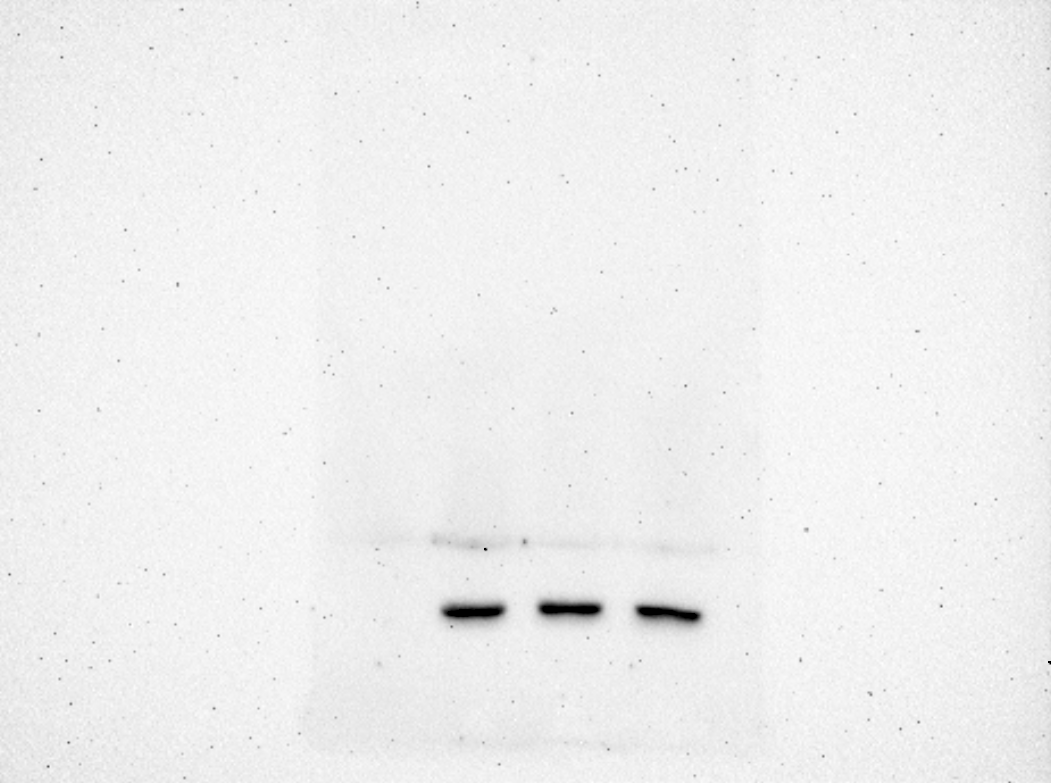

Supplement: Figure 3—source data 3. — Whole-cell lysates (PRE-IP) of MC38 cells was used as control of protein expression of IDO1, Src, and β-actin. The negative control (i.e., sample not immunoprecipitated with the antibody) is included. Figure with the uncropped blots with relevant bands clearly labeled are provided. [file elife-85872-fig3-data3.zip › Figure 3-source data 9/PRE IP_a-IDO1.tif]

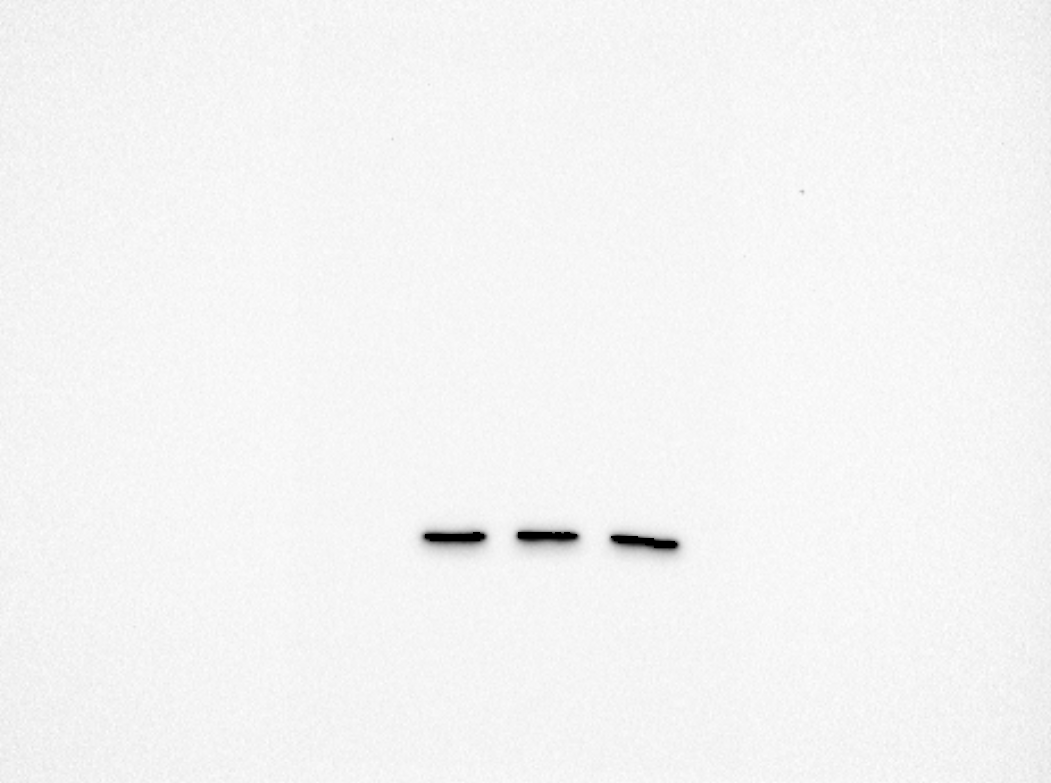

Supplement: Figure 3—source data 3. — Whole-cell lysates (PRE-IP) of MC38 cells was used as control of protein expression of IDO1, Src, and β-actin. The negative control (i.e., sample not immunoprecipitated with the antibody) is included. Figure with the uncropped blots with relevant bands clearly labeled are provided. [file elife-85872-fig3-data3.zip › Figure 3-source data 9/PRE IP_a-Src.tif]

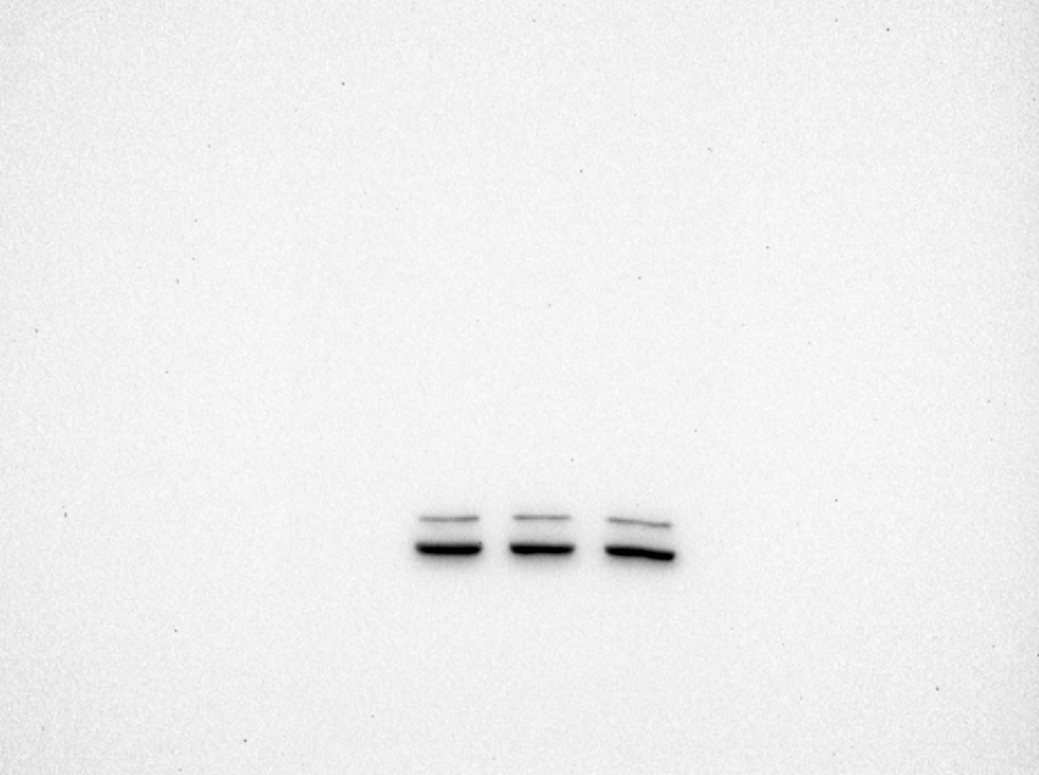

Supplement: Figure 3—source data 3. — Whole-cell lysates (PRE-IP) of MC38 cells was used as control of protein expression of IDO1, Src, and β-actin. The negative control (i.e., sample not immunoprecipitated with the antibody) is included. Figure with the uncropped blots with relevant bands clearly labeled are provided. [file elife-85872-fig3-data3.zip › Figure 3-source data 9/PRE IP_a-tubulin.tif]

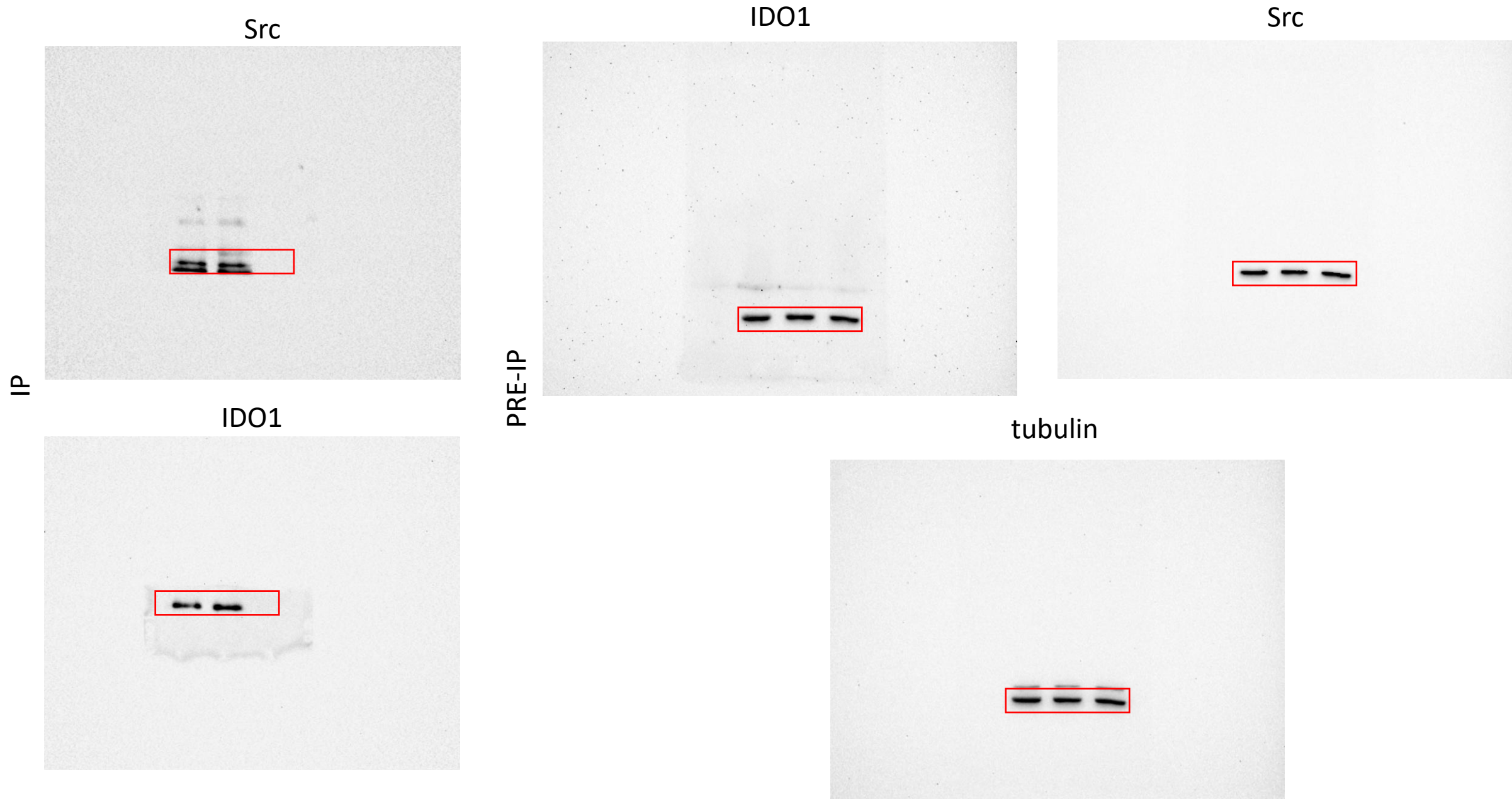

Supplement: Figure 3—source data 3. — Whole-cell lysates (PRE-IP) of MC38 cells was used as control of protein expression of IDO1, Src, and β-actin. The negative control (i.e., sample not immunoprecipitated with the antibody) is included. Figure with the uncropped blots with relevant bands clearly labeled are provided. [file elife-85872-fig3-data3.zip › Figure 3-source data 9/Rossini et al. Figure 3E_uncropped.pdf]

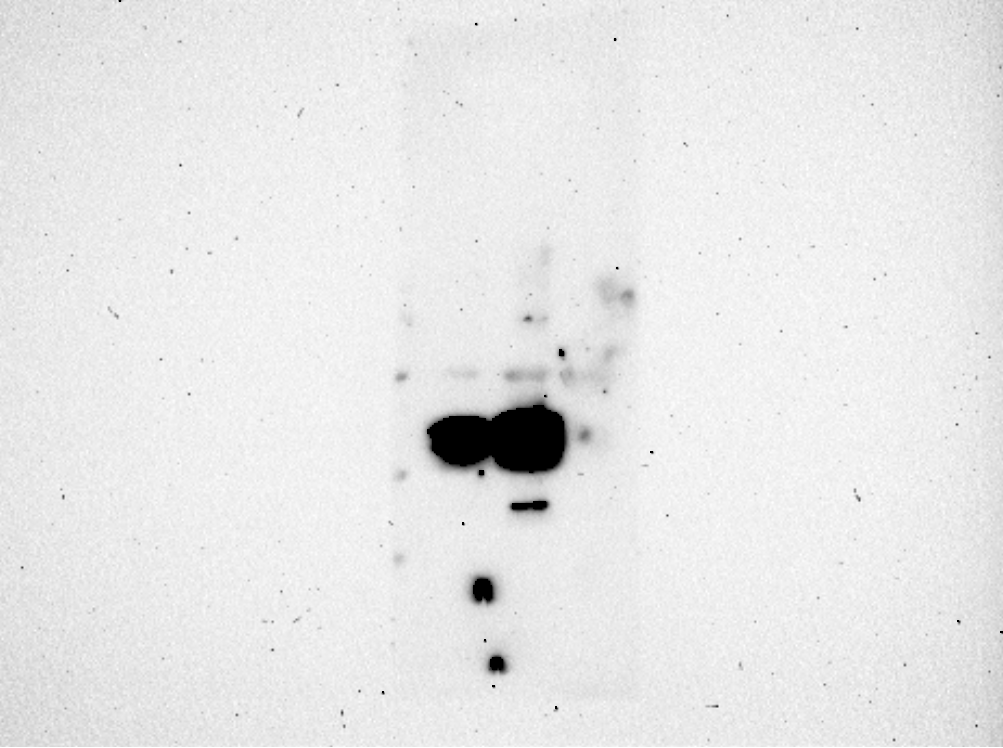

Supplement: Figure 3—source data 4. — The detection of indoleamine 2,3-dioxygenase 1 (IDO1) and Src was performed by sequential immunoblotting with specific antibodies (IP). Whole-cell lysates (PRE-IP) was used as control of protein expression. The negative control (i.e., sample not immunoprecipitated with the antibody) is included. Figure with the uncropped blots with relevant bands clearly labeled are provided. [file elife-85872-fig3-data4.zip › Figure 3-source data 10/IP-apTYR-a-IDO1.tif]

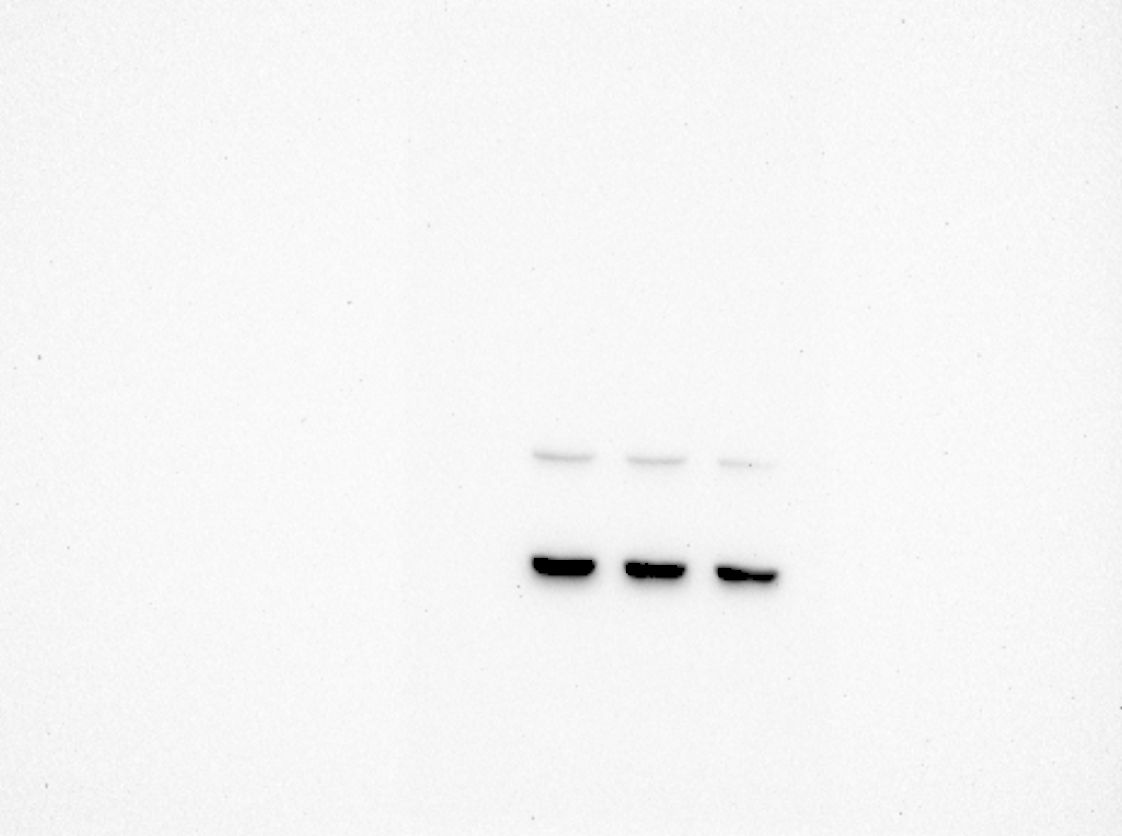

Supplement: Figure 3—source data 4. — The detection of indoleamine 2,3-dioxygenase 1 (IDO1) and Src was performed by sequential immunoblotting with specific antibodies (IP). Whole-cell lysates (PRE-IP) was used as control of protein expression. The negative control (i.e., sample not immunoprecipitated with the antibody) is included. Figure with the uncropped blots with relevant bands clearly labeled are provided. [file elife-85872-fig3-data4.zip › Figure 3-source data 10/PRE IP_a-Actin.tif]

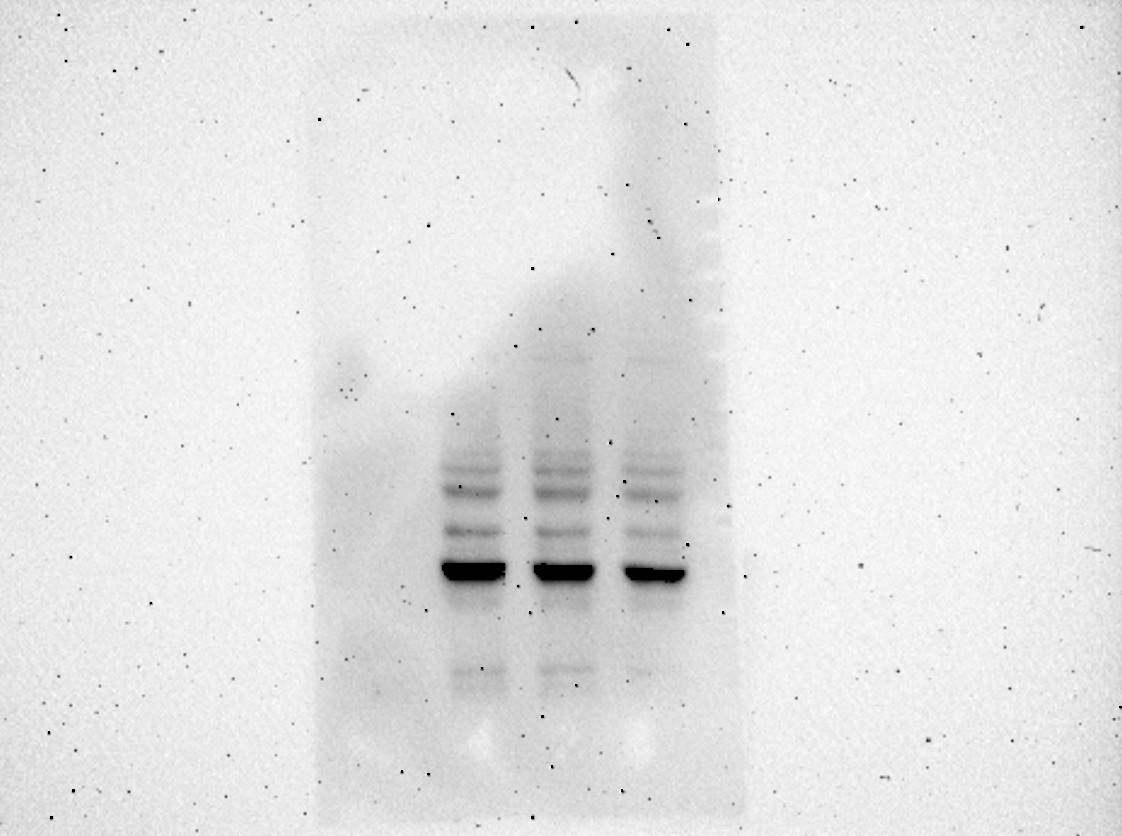

Supplement: Figure 3—source data 4. — The detection of indoleamine 2,3-dioxygenase 1 (IDO1) and Src was performed by sequential immunoblotting with specific antibodies (IP). Whole-cell lysates (PRE-IP) was used as control of protein expression. The negative control (i.e., sample not immunoprecipitated with the antibody) is included. Figure with the uncropped blots with relevant bands clearly labeled are provided. [file elife-85872-fig3-data4.zip › Figure 3-source data 10/PRE IP_a-IDO1.tif]

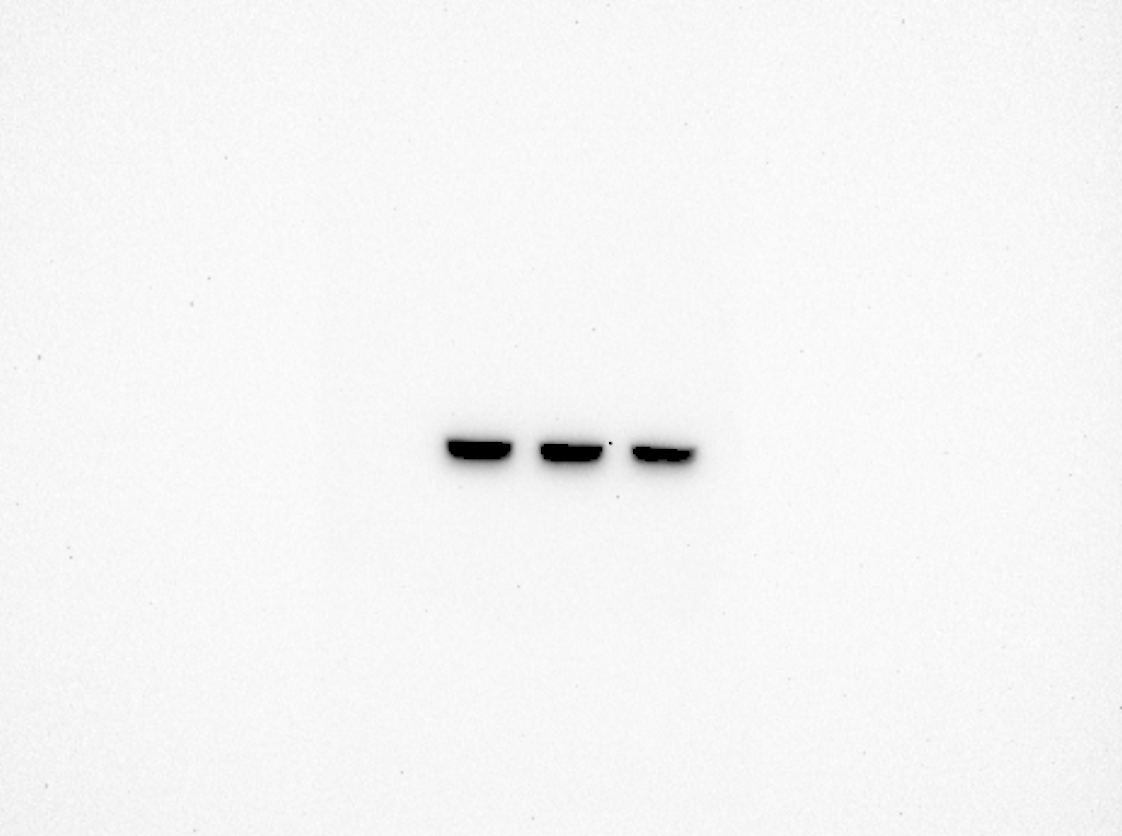

Supplement: Figure 3—source data 4. — The detection of indoleamine 2,3-dioxygenase 1 (IDO1) and Src was performed by sequential immunoblotting with specific antibodies (IP). Whole-cell lysates (PRE-IP) was used as control of protein expression. The negative control (i.e., sample not immunoprecipitated with the antibody) is included. Figure with the uncropped blots with relevant bands clearly labeled are provided. [file elife-85872-fig3-data4.zip › Figure 3-source data 10/PRE IP_a-Src.tif]

PRE-IP

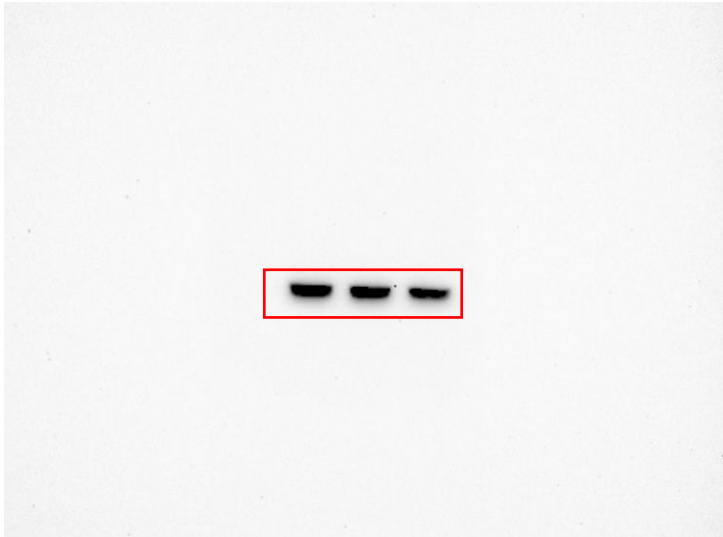

Src

IP

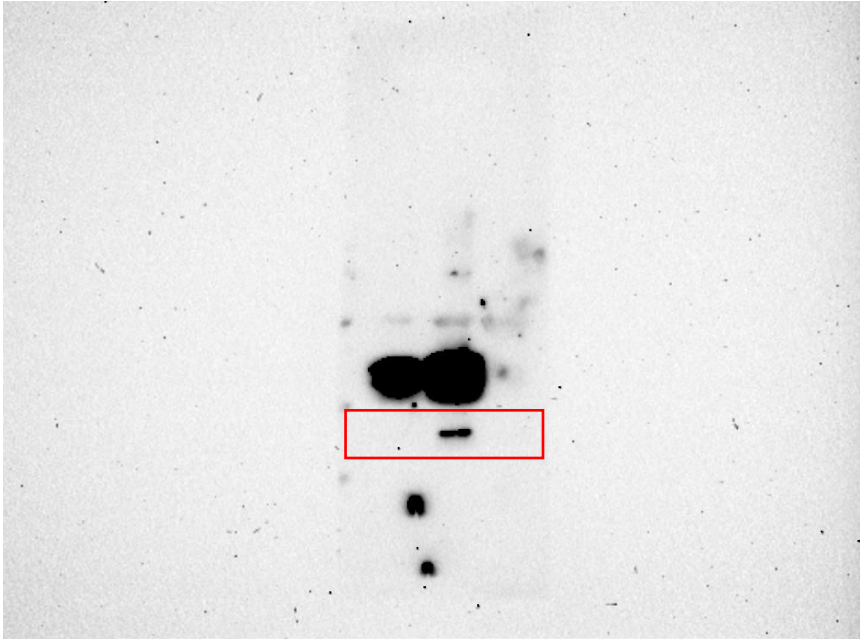

IDO1

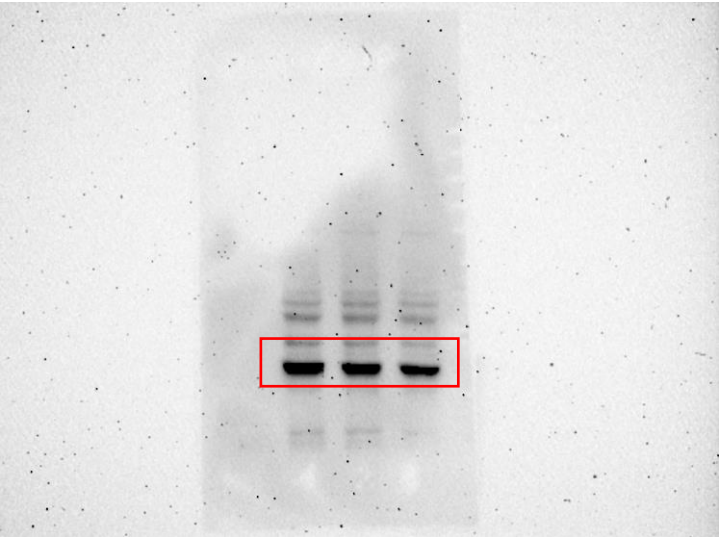

Actin

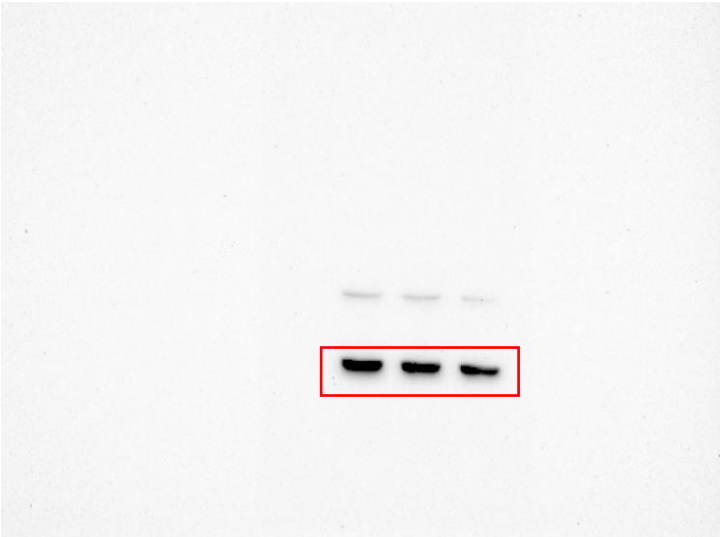

Supplement: Figure 3—source data 4. — The detection of indoleamine 2,3-dioxygenase 1 (IDO1) and Src was performed by sequential immunoblotting with specific antibodies (IP). Whole-cell lysates (PRE-IP) was used as control of protein expression. The negative control (i.e., sample not immunoprecipitated with the antibody) is included. Figure with the uncropped blots with relevant bands clearly labeled are provided. [file elife-85872-fig3-data4.zip › Figure 3-source data 10/Rossini et al. Figure 3H_uncropped.pdf]

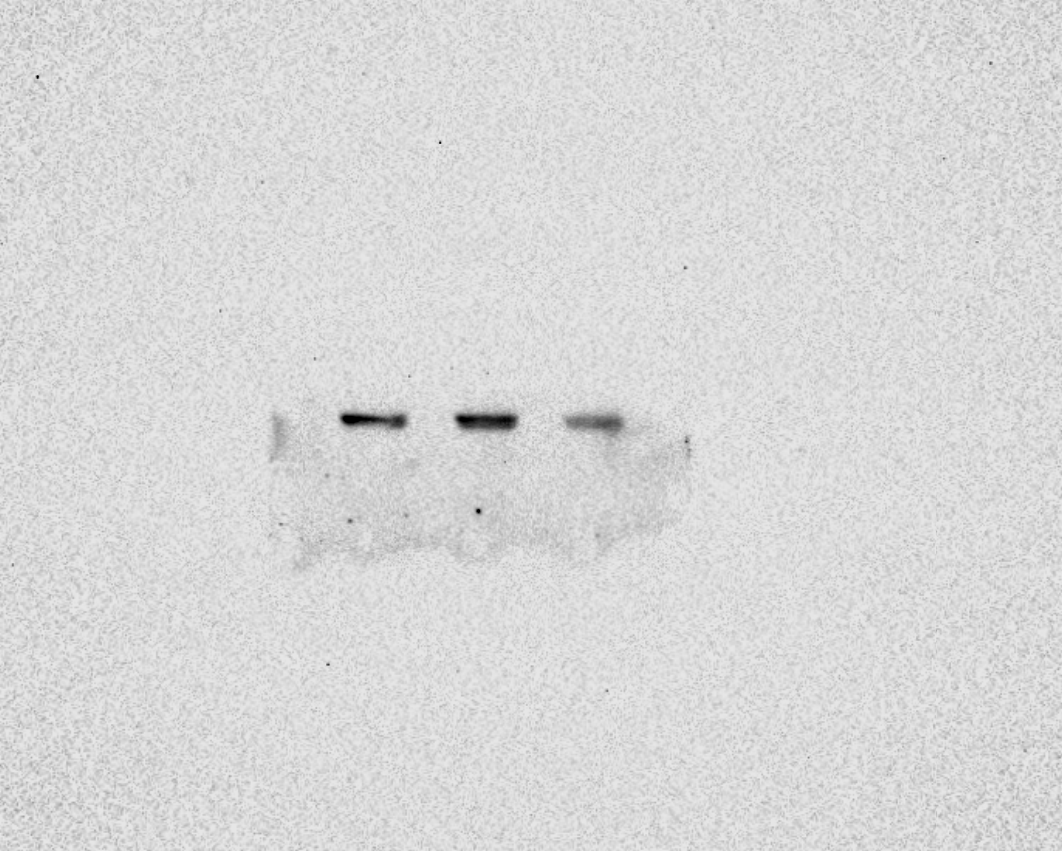

Supplement: Figure 3—source data 5. — The detection of indoleamine 2,3-dioxygenase 1 (IDO1) and Src was performed by sequential immunoblotting with specific antibodies (IP). Whole-cell lysates (PRE-IP) was used as control of protein expression. The negative control (i.e., sample not immunoprecipitated with the antibody) is included. Figure with the uncropped blots with relevant bands clearly labeled are provided. [file elife-85872-fig3-data5.zip › Figure 3-source data 11/IP_aIDO1.tif]

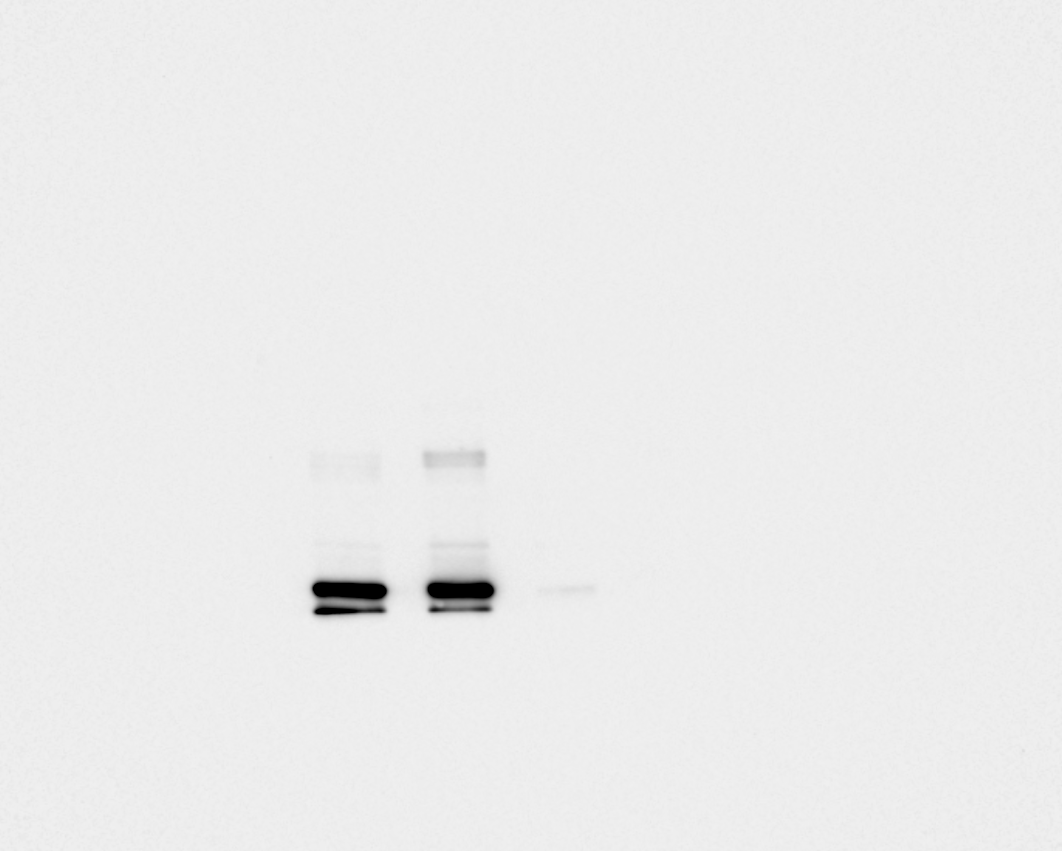

Supplement: Figure 3—source data 5. — The detection of indoleamine 2,3-dioxygenase 1 (IDO1) and Src was performed by sequential immunoblotting with specific antibodies (IP). Whole-cell lysates (PRE-IP) was used as control of protein expression. The negative control (i.e., sample not immunoprecipitated with the antibody) is included. Figure with the uncropped blots with relevant bands clearly labeled are provided. [file elife-85872-fig3-data5.zip › Figure 3-source data 11/IP_aSrc.tif]

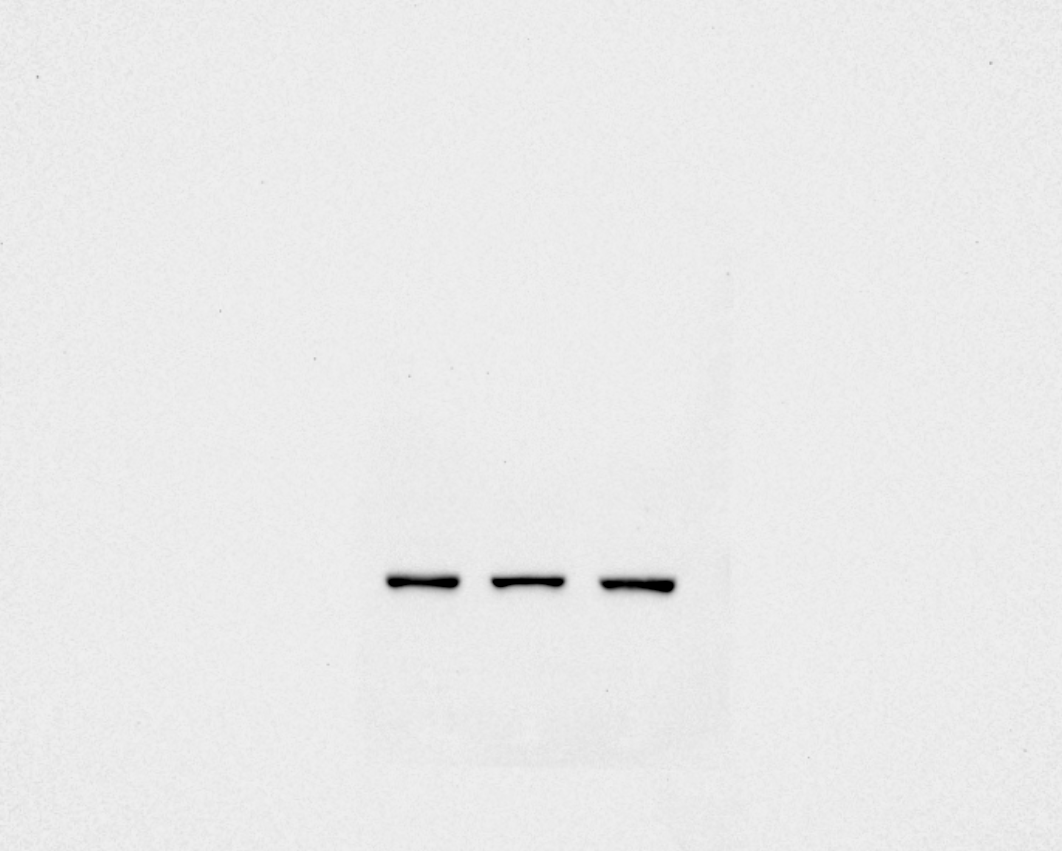

Supplement: Figure 3—source data 5. — The detection of indoleamine 2,3-dioxygenase 1 (IDO1) and Src was performed by sequential immunoblotting with specific antibodies (IP). Whole-cell lysates (PRE-IP) was used as control of protein expression. The negative control (i.e., sample not immunoprecipitated with the antibody) is included. Figure with the uncropped blots with relevant bands clearly labeled are provided. [file elife-85872-fig3-data5.zip › Figure 3-source data 11/PRE IP-aIDO1.tif]

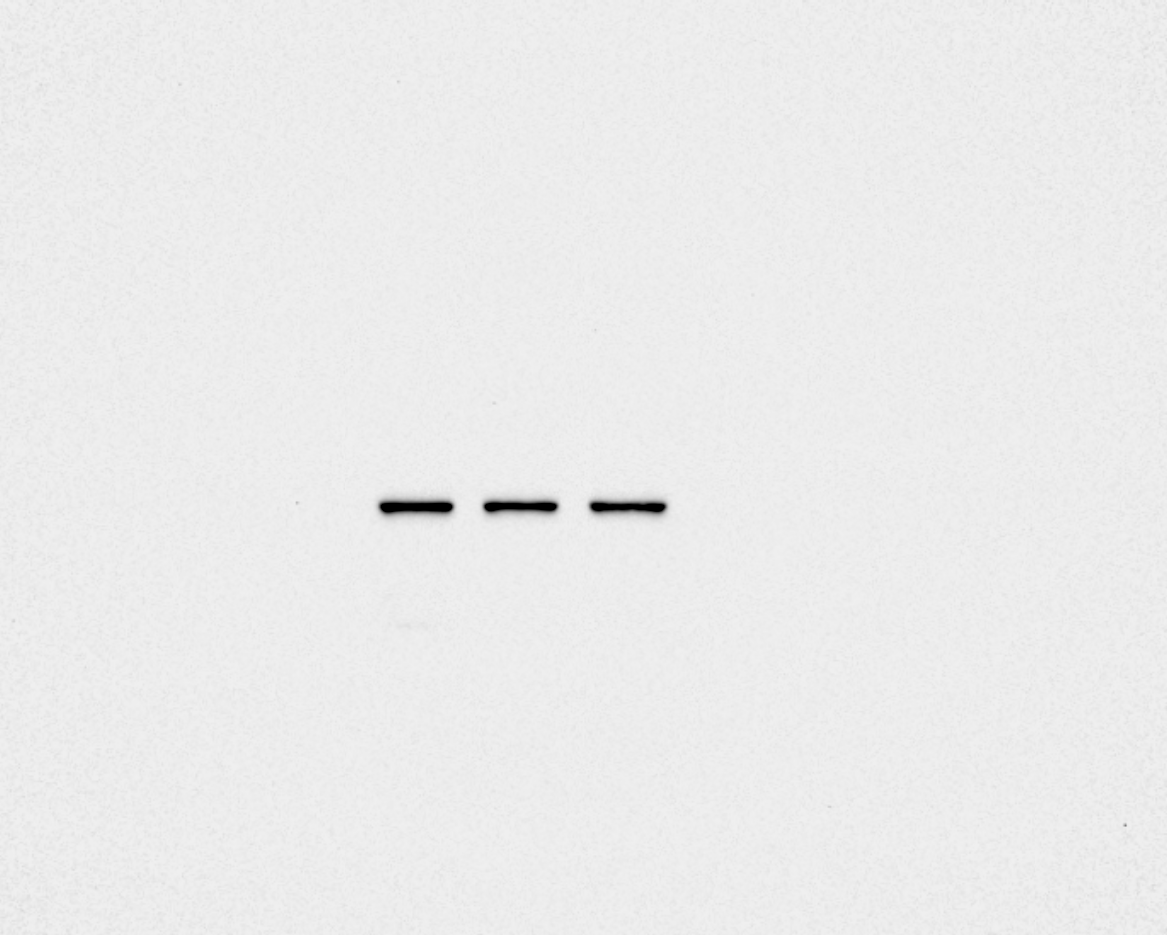

Supplement: Figure 3—source data 5. — The detection of indoleamine 2,3-dioxygenase 1 (IDO1) and Src was performed by sequential immunoblotting with specific antibodies (IP). Whole-cell lysates (PRE-IP) was used as control of protein expression. The negative control (i.e., sample not immunoprecipitated with the antibody) is included. Figure with the uncropped blots with relevant bands clearly labeled are provided. [file elife-85872-fig3-data5.zip › Figure 3-source data 11/PRE IP-aSrc.tif]

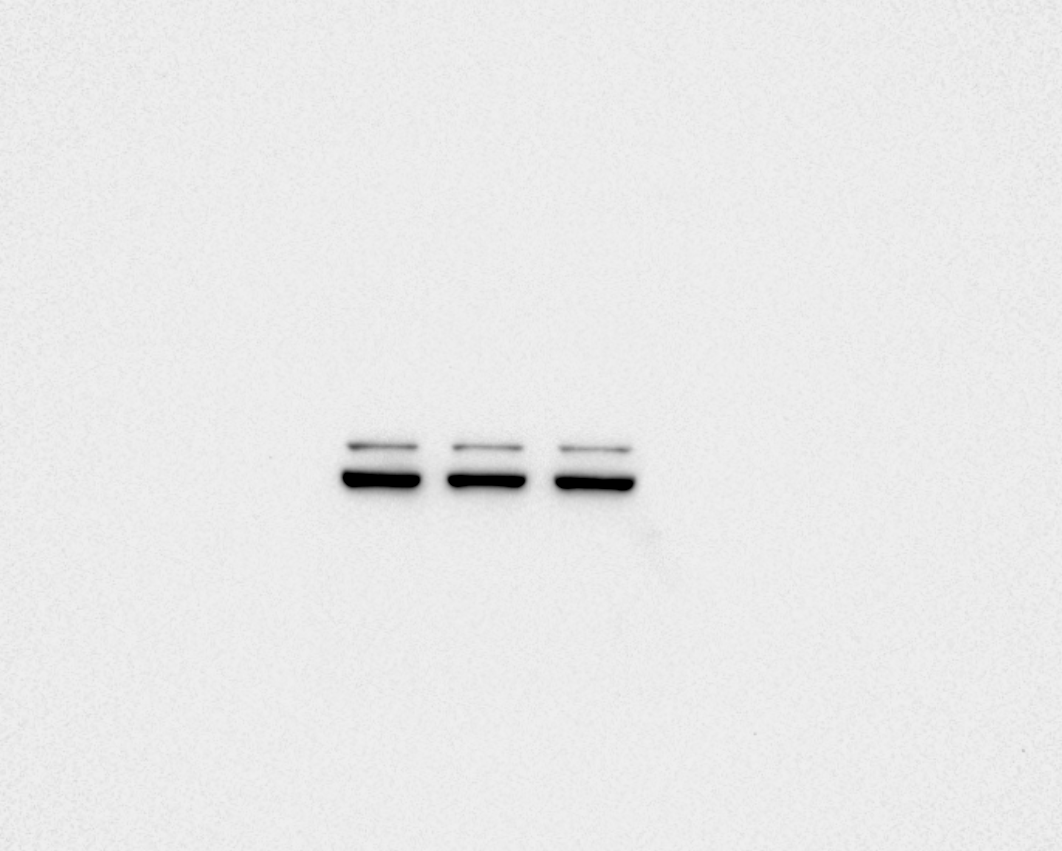

Supplement: Figure 3—source data 5. — The detection of indoleamine 2,3-dioxygenase 1 (IDO1) and Src was performed by sequential immunoblotting with specific antibodies (IP). Whole-cell lysates (PRE-IP) was used as control of protein expression. The negative control (i.e., sample not immunoprecipitated with the antibody) is included. Figure with the uncropped blots with relevant bands clearly labeled are provided. [file elife-85872-fig3-data5.zip › Figure 3-source data 11/PRE IP-atubulin.tif]

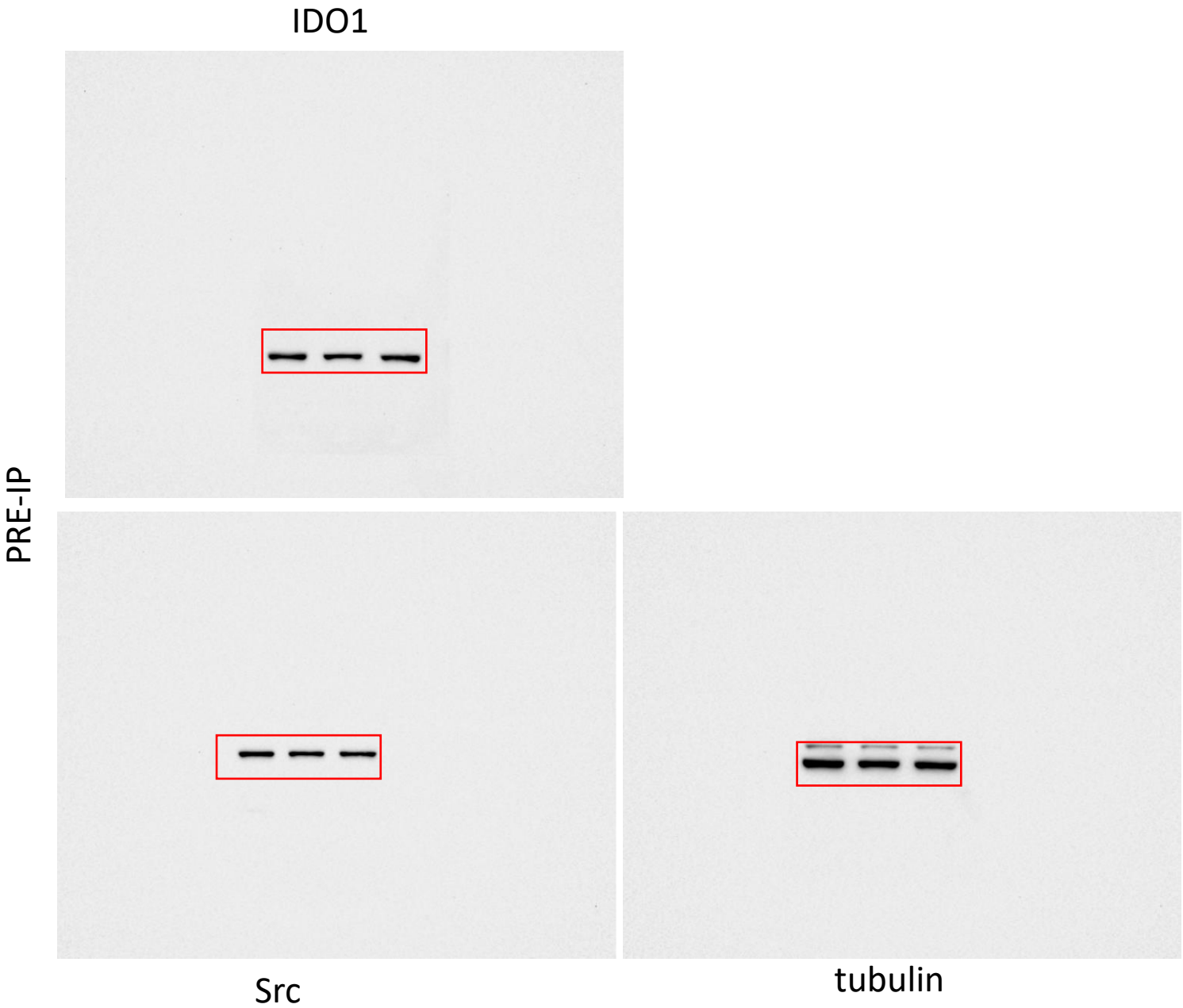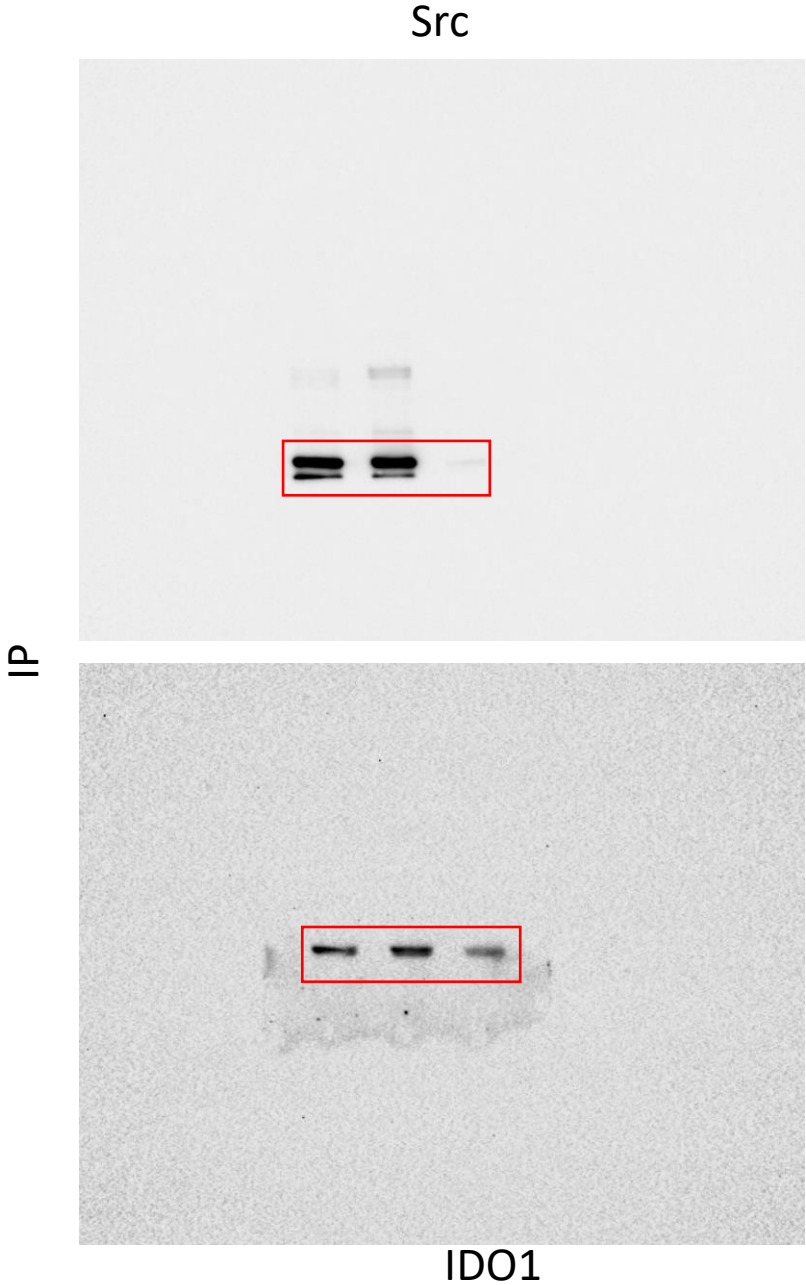

Supplement: Figure 3—source data 5. — The detection of indoleamine 2,3-dioxygenase 1 (IDO1) and Src was performed by sequential immunoblotting with specific antibodies (IP). Whole-cell lysates (PRE-IP) was used as control of protein expression. The negative control (i.e., sample not immunoprecipitated with the antibody) is included. Figure with the uncropped blots with relevant bands clearly labeled are provided. [file elife-85872-fig3-data5.zip › Figure 3-source data 11/Rossini et al. Figure 3I_uncropped.pdf]

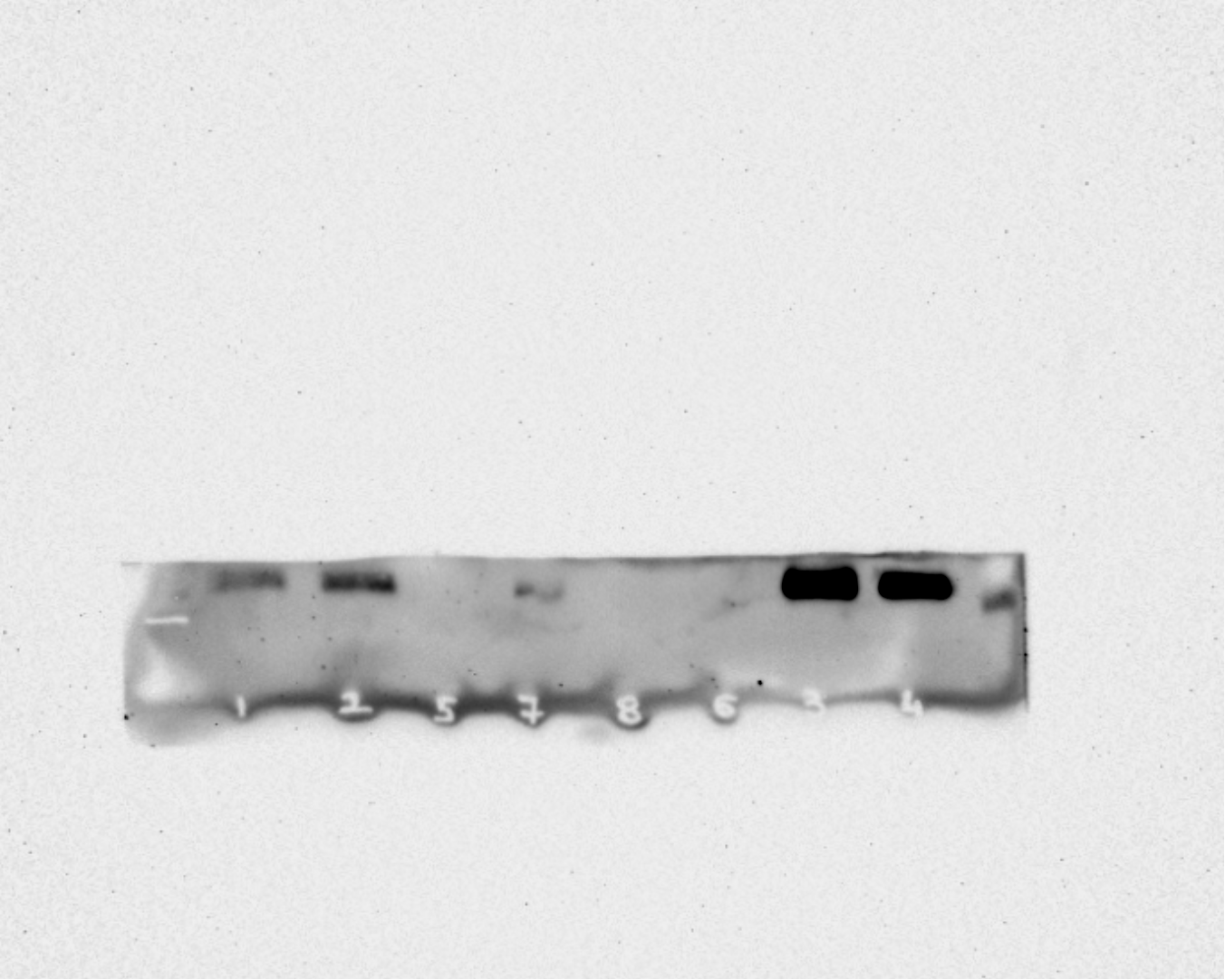

Supplement: Figure 3—figure supplement 1—source data 1. — Whole-cell lysates (PRE-IP) was used as control of protein expression of IDO1, Src, and β-tubulin. SYF cells were reconstituted with vectors coding for wild-type Src and IDO1 or Src mutated at tyrosine 529 with phenylalanine and IDO1. Moreover, cells transfected with vectors coding for either Src or IDO1 were used for the experiments. The negative control (i.e., sample expressing both IDO1 and Src, but not immunoprecipitated with the antibody) is included. Cells were either treated with spermidine (100 μM) or left untreated. Figure with the uncropped blots with relevant bands clearly labeled are provided. [file elife-85872-fig3-figsupp1-data1.zip › Figure 3 - Figure Supplement 1 - Source Data 12/IP-aIDO1.tif]

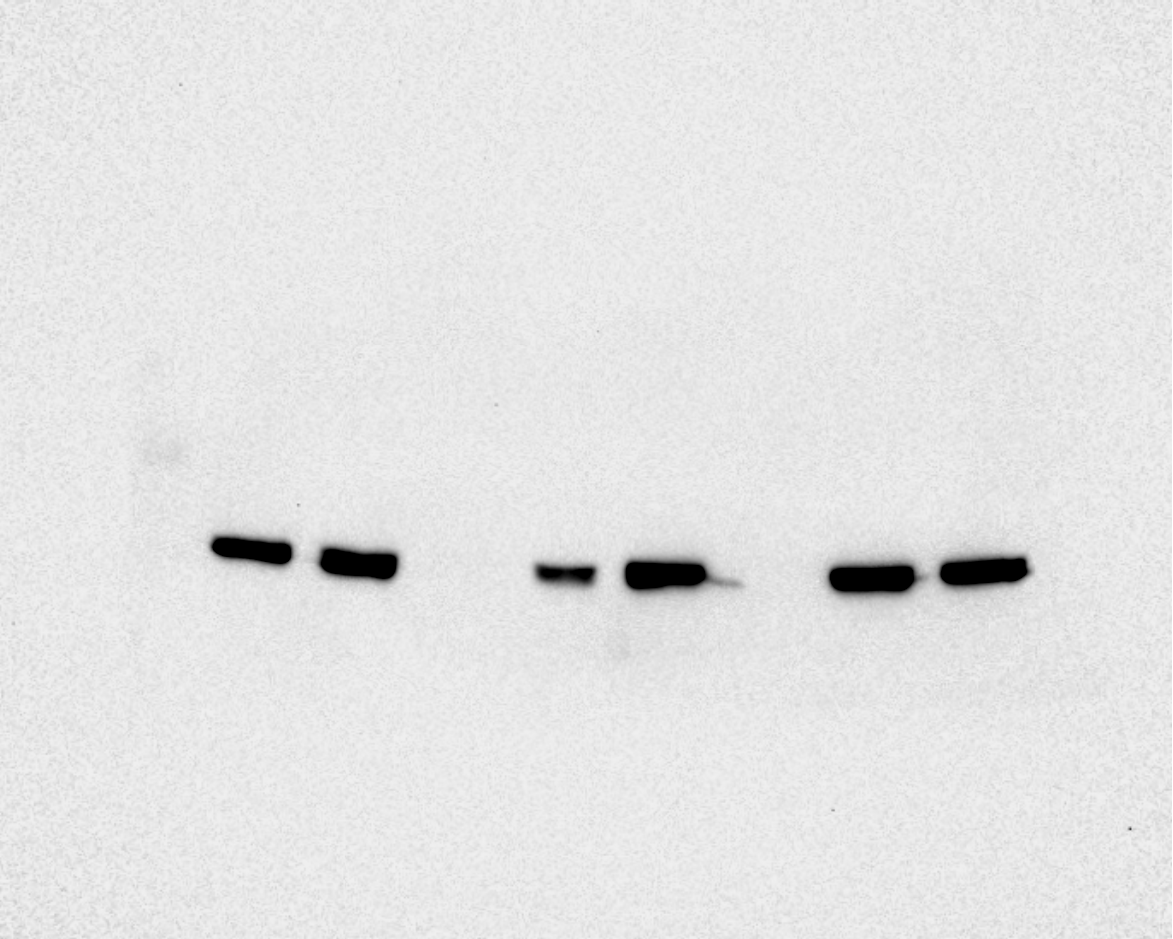

Supplement: Figure 3—figure supplement 1—source data 1. — Whole-cell lysates (PRE-IP) was used as control of protein expression of IDO1, Src, and β-tubulin. SYF cells were reconstituted with vectors coding for wild-type Src and IDO1 or Src mutated at tyrosine 529 with phenylalanine and IDO1. Moreover, cells transfected with vectors coding for either Src or IDO1 were used for the experiments. The negative control (i.e., sample expressing both IDO1 and Src, but not immunoprecipitated with the antibody) is included. Cells were either treated with spermidine (100 μM) or left untreated. Figure with the uncropped blots with relevant bands clearly labeled are provided. [file elife-85872-fig3-figsupp1-data1.zip › Figure 3 - Figure Supplement 1 - Source Data 12/PREIP-aIDO1.tif]

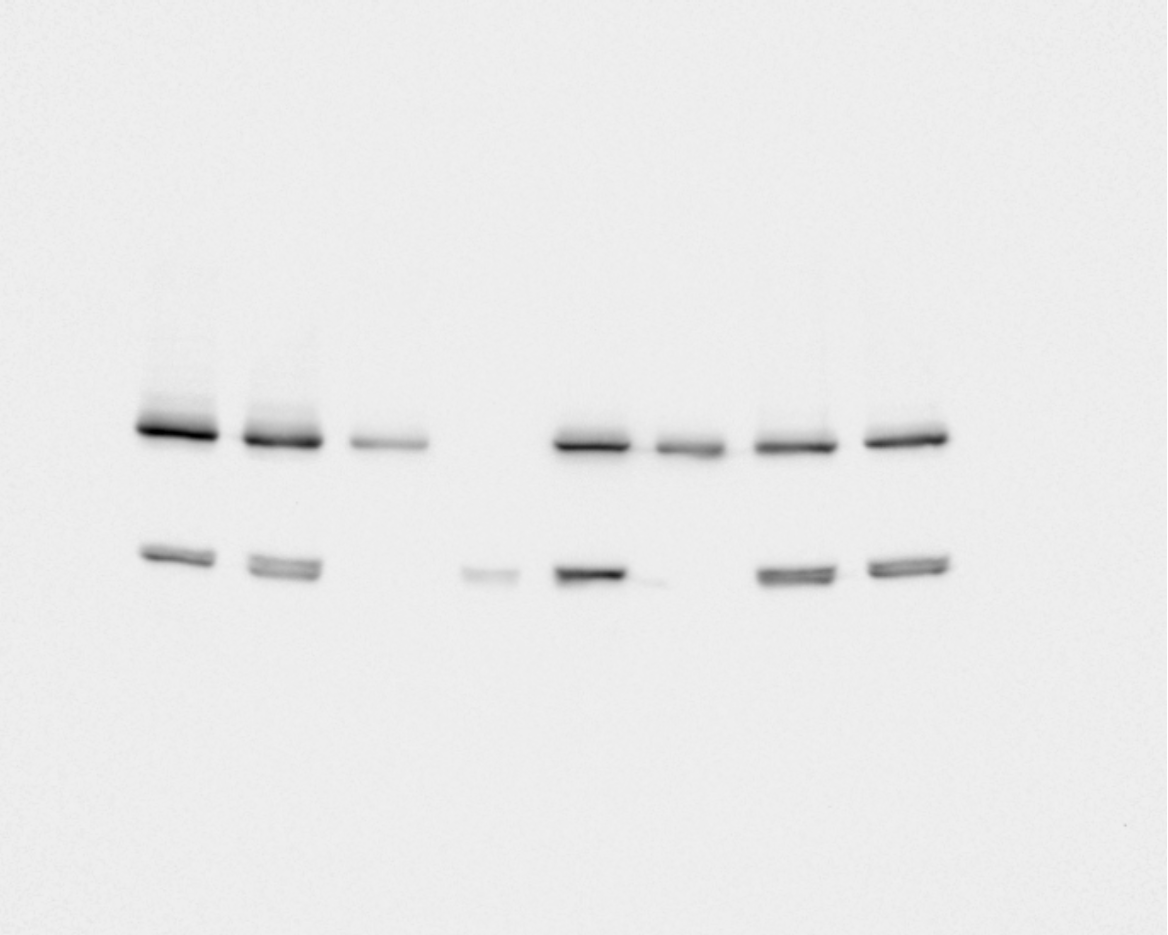

Supplement: Figure 3—figure supplement 1—source data 1. — Whole-cell lysates (PRE-IP) was used as control of protein expression of IDO1, Src, and β-tubulin. SYF cells were reconstituted with vectors coding for wild-type Src and IDO1 or Src mutated at tyrosine 529 with phenylalanine and IDO1. Moreover, cells transfected with vectors coding for either Src or IDO1 were used for the experiments. The negative control (i.e., sample expressing both IDO1 and Src, but not immunoprecipitated with the antibody) is included. Cells were either treated with spermidine (100 μM) or left untreated. Figure with the uncropped blots with relevant bands clearly labeled are provided. [file elife-85872-fig3-figsupp1-data1.zip › Figure 3 - Figure Supplement 1 - Source Data 12/PREIP-a-SRC.tif]

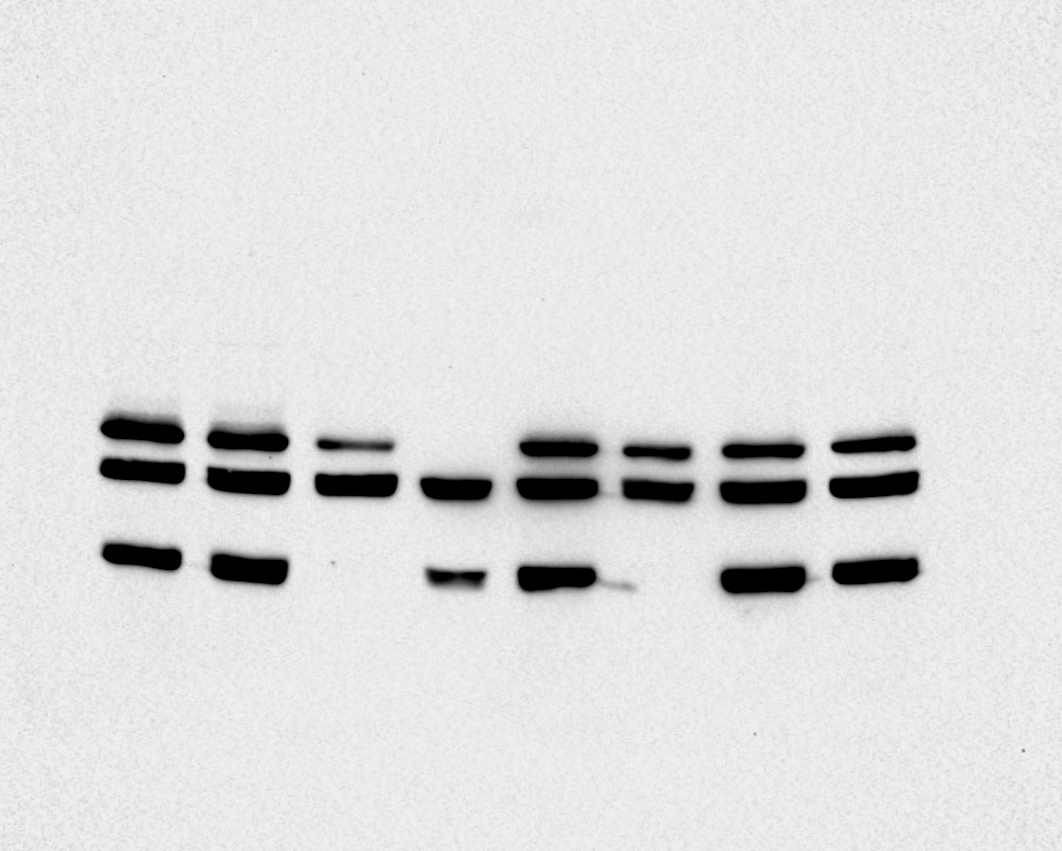

Supplement: Figure 3—figure supplement 1—source data 1. — Whole-cell lysates (PRE-IP) was used as control of protein expression of IDO1, Src, and β-tubulin. SYF cells were reconstituted with vectors coding for wild-type Src and IDO1 or Src mutated at tyrosine 529 with phenylalanine and IDO1. Moreover, cells transfected with vectors coding for either Src or IDO1 were used for the experiments. The negative control (i.e., sample expressing both IDO1 and Src, but not immunoprecipitated with the antibody) is included. Cells were either treated with spermidine (100 μM) or left untreated. Figure with the uncropped blots with relevant bands clearly labeled are provided. [file elife-85872-fig3-figsupp1-data1.zip › Figure 3 - Figure Supplement 1 - Source Data 12/PREIP-aTUB.tif]

IDO1  
IP

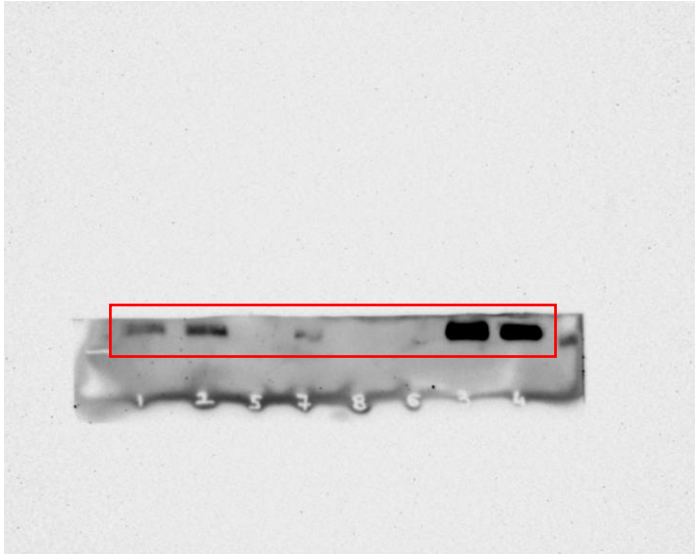

IDO

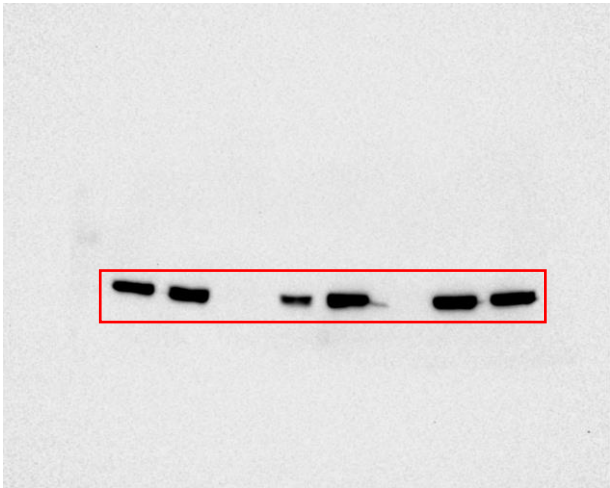

Src

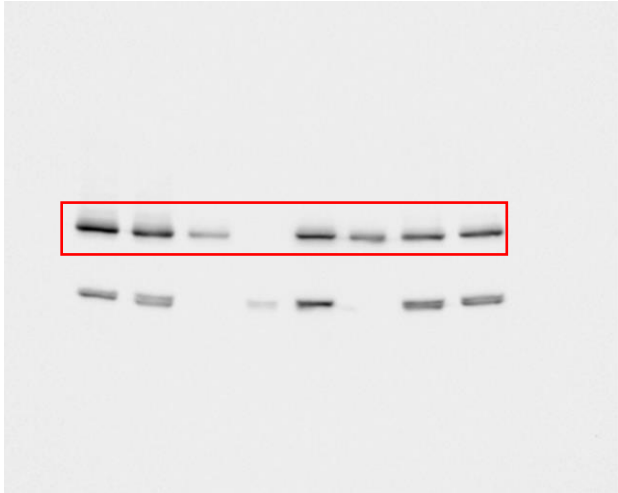

PRE-IP

tubulin

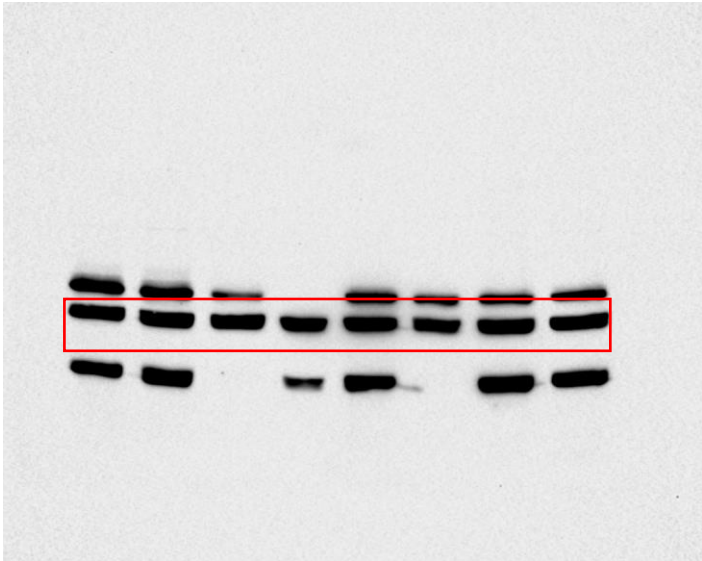

Supplement: Figure 3—figure supplement 1—source data 1. — Whole-cell lysates (PRE-IP) was used as control of protein expression of IDO1, Src, and β-tubulin. SYF cells were reconstituted with vectors coding for wild-type Src and IDO1 or Src mutated at tyrosine 529 with phenylalanine and IDO1. Moreover, cells transfected with vectors coding for either Src or IDO1 were used for the experiments. The negative control (i.e., sample expressing both IDO1 and Src, but not immunoprecipitated with the antibody) is included. Cells were either treated with spermidine (100 μM) or left untreated. Figure with the uncropped blots with relevant bands clearly labeled are provided. [file elife-85872-fig3-figsupp1-data1.zip › Figure 3 - Figure Supplement 1 - Source Data 12/Rossini et al. Figure 3-Supplement figure 1_uncropped.pdf]
