# Supplementary material for: A back-door insight into the modulation of Src kinase activity by the polyamine spermidine
Source: eLife. 2023 Jun 30;12:e85872. doi: 10.7554/eLife.85872 (PMC10328509; doi:10.7554/eLife.85872)
Supplement: Supplementary file 1. [file elife-85872-supp1.docx]

| **Pose #** | **IFD Score (kcal/mol)** | **Δ IFD Score (kcal/mol)** |
| --- | --- | --- |
| 1 | -227.50 | 0.00 |
| 2 | -227.41 | +0.08 |
| 3 | -227.11 | +0.39 |
| 4 | -227.03 | +0.47 |
| 5 | -227.00 | +0.50 |
| 6 | -226.95 | +0.55 |
| 7 | -226.70 | +0.79 |
| 8 | -226.68 | +0.81 |
| 9 | -226.68 | +0.82 |
| 10 | -226.66 | +0.83 |
| 11 | -226.58 | +0.92 |
| 12 | -226.56 | +0.94 |
| 13 | -226.54 | +0.95 |
| 14 | -226.49 | +1.01 |
| 15 | -226.43 | +1.07 |
| 16 | -226.34 | +1.15 |
| 17 | -226.15 | +1.34 |
| 18 | -225.31 | +2.19 |
